# Spontaneously fermented *Allium cepa* L. as a source of lactic acid bacteria with probiotic potential

**DOI:** 10.1038/s41598-025-10037-7

**Published:** 2025-07-17

**Authors:** Sylwia Ścieszka, Lidia Piekarska-Radzik, Robert Klewicki, Michał Sójka, Jerzy Juśkiewicz, Bartosz Fotschki, Elżbieta Klewicka, Katarzyna Grzelak-Błaszczyk

**Affiliations:** 1 Institute of Fermentation Technology and Microbiology, Lodz University of Technology, Wólczańska 171/173, Łódź, 90-530 Poland; 2 Institute of Food Technology and Analysis, Lodz University of Technology, Stefanowskiego 2/22, Łódź, 90-537 Poland; 3 Institute of Animal Reproduction and Food Research, Polish Academy of Sciences, Trylińskiego 18, Olsztyn, 10-683 Poland

**Keywords:** Fermented onions, Lactic acid bacteria, Probiotic, Antimicrobial activity, Biotechnology, Microbiology, Molecular biology, Physiology, Plant sciences

## Abstract

Onions (*Allium cepa* L.) are widely recognized for their nutritional and medicinal properties. This study investigates the suitability of spontaneously fermented onions as a natural source of lactic acid bacteria (LAB) with probiotic potential. The isolated LAB were identified as *Lactiplantibacillus plantarum*,* Lactiplantibacillus pentosus*, and *Levilactobacillus brevis*. Comprehensive analysis were conducted to evaluate their ability to ferment various carbohydrates, enzymatic activity, resistance to environmental stressors (pH, bile salts, NaCl, H₂O₂, and phenol), and antimicrobial properties. Most strains demonstrated high survivability in acidic conditions (pH 3.0) and bile salts (0.4%), crucial for probiotic viability in the gastrointestinal tract, and exhibited antimicrobial activity against foodborne pathogens. These antimicrobial effects were primarily due to organic acid production, with hydrogen peroxide synthesis providing additional support. Additionally, none of the tested strains hydrolyzed mucin, confirming their safety for potential probiotic applications. These findings indicate that LAB from fermented onions could be promising candidates for application in probiotic and functional food formulations.

**Supplementary Information:**

The online version contains supplementary material available at 10.1038/s41598-025-10037-7.

## Introduction

Onion (*Allium cepa* L.) belongs to the *Amaryllidaceae* family and is one of the most widespread vegetables in the world. Onions have been known since antiquity as both a food and a valuable medicinal product. In various cultures, they have been used to treat numerous ailments, including bacterial and fungal infections, respiratory diseases, gastrointestinal disorders, skin conditions, cardiovascular issues, diabetes, renal colic, rheumatism, menstrual pain, and headaches^1^. In 2021, global onion production was 106.59 M tons, with India producing 26.64 M tons, China 24.16 M tons, Egypt 3.312 M tons, the United States 3.10 M tons, Türkiye 2.50 M tons, and Pakistan 2.30 M tons^2^. In 2022, the leading onion producers in the European Union were the Netherlands (1.5 M tons), Spain (1.22 M tons), France (0.71 M tons), Poland (0.64 M tons), and Germany (0.64 M tons) (Eurostat, 2024).

The nutritional composition of *Allium cepa* L. includes 89% water, 4% saccharides, about 2% dietary fiber, 1% protein, and only 0.1% fat^3,4^. In terms of biologically active compounds, onions are rich in ferulic acid, gallic acid, protocatechuic acid, quercetin, kaempferol, anthocyanins, and dipropyl disulfide and trisulfide as the main components of onion oil^5–7^. The content of these compounds can vary depending on the onion variety. However, onions are considered one of the best sources of quercetin, with concentrations ranging from 14.5 to 5110 µg/g depending on the variety^6^.

Saccharides and fructo-oligosaccharides (FOSs) are important nutrients in onions. The monosaccharides found in onion bulbs include glucose and fructose, while disaccharides include sucrose. Onions also contain low-molecular-weight fructo-oligosaccharides (FOSs: 1-kestose, neokestose, nystose), linear fructans (inulin), and branched fructans (inulin neoseries polymers)^8–11^. The presence of FOSs in food is highly desirable due to their prebiotic effects, as they can be metabolized by bacterial enzymes, particularly those from lactic acid bacteria (LAB) from the *Lactobacillaceae* family and bacteria belonging to the *Bifidobacterium* genus^12^. Due to the presence of FOSs, onions are an excellent substrate for the growth of LAB. During controlled fermentation of onion bulbs, LAB from the species *Levilactobacillus brevis* reached abundances of 7.6–9.4 log CFU/mL in the liquid after fermentation^13^. LAB account for approximately 10²–10⁴ CFU/g of the autochthonous microflora in vegetables and fruits^14,15^. In the case of *Lactobacillaceae* strains, this constitutes around 20% of the initial number of bacteria involved in spontaneous fermentation. During spontaneous fermentation, LAB can dominate the environment, reaching levels of 10⁷ CFU/mL^16^.

Modern food processing focuses increasingly on the development of functional, probiotic, and prebiotic foods. The onion (*Allium cepa* L.) serves as an excellent raw material for such purposes. However, producing a fermented product with consistent characteristics requires the use of LAB strains that are well-adapted to the onion matrix, preferably those originating from the same environment. These strains should quickly adapt to the environment, avoid growth inhibition by phytochemicals, and efficiently utilize the plant’s nutrients. Therefore, LAB isolated from spontaneously fermented vegetables and fruits seem to be promising strains with probiotic potential. Nevertheless, these strains must be validated according to the FAO/WHO definition of probiotics^17^. Their functional properties, species identification, survival at low pH and in the presence of bile salts, antimicrobial activity, and antibiotic resistance profiles need to be evaluated.

The aim of this study was to obtain new strains of LAB and characterize them in terms of their probiotic properties and technological applications. These strains are expected to play a crucial role in the development of innovative formulations for osmotically dehydrated, fermented onions, representing a significant advancement in food processing technology.

## Materials and methods

### Plant material

The plant material consisted of yellow onions (7 samples), red onions (2 samples), and shallot onion (1 sample). The onions were purchased from ALDI (ALDI Nord Group, Germany) stores in Aleksandrów Łódzki (Poland), Stokrotka (Maxima, Lithuania) store in Łódź (Poland), POLOmarket in Jastrzębia Góra (Poland), and local vegetable markets in Jastrzębia Góra, Poddębice, Pabianice (Poland).

### Spontaneous fermentation of common onions

Onions were peeled from dry skins and the first layer of fresh skins. The peeled onions were then cut into smaller pieces of 4–6 portions (approximately 5–7 cm in length, 2–3 cm in width, and 1.5–2.0 cm in thickness). Approximately 100 g of raw onion was placed in PYREX glass bottles and poured with 1% (*w/v*) sterile sodium chloride (Sigma-Aldrich, Darmstadt, Germany) to cover the entire onion (70–100 mL). The samples were incubated for 10 to 14 days at 30 °C (Fig. S1). After this time, the fermentation liquid was collected, and the isolation of LAB was started.

### Isolation of LAB

One mL of fermentation liquid was inoculated in 9 mL of MRS Broth medium (Merck, Darmstadt, Germany) and incubated for 48 h under the following conditions, optimal for LAB growth: 30 °C, 37 °C, 37 °C in the presence of 5% (*v/v*) CO_2_ using a CO_2_ chamber (S@fegrow 188 PRO, EUROCLONE S.p.A, Pero, Italy). After this time, bacterial growth was evaluated by measuring the optical density (OD) at 600 nm. Fermentation was terminated when OD₆₀₀ exceeded 1.0. Samples were then plated onto MRS Agar medium (Merck, Darmstadt, Germany) using the reduction method and incubated under the same conditions as the original samples for 48 h. Then, the ability to synthesize catalase of individual colonies was assessed. Catalase-negative colonies were selected for further analysis. Gram staining was performed to confirm Gram-positive morphology. Selected colonies were rescreened reductively to obtain single colonies. Pure cultures obtained from single colonies were propagated, and cryobanks (Mast Diagnostica GmbH, Reinfeld, Germany) were prepared. The cryobanks were stored at -20 °C.

### Identification of isolated LAB strains

#### Identification based on fermentation profile

The carbohydrate fermentation profile of the LAB isolates was determined using API 50 CHL (BioMérieux, Marcy l’Etoile, France) following the manufacturer’s instructions. The results were compared with the online database (https://apiweb.biomerieux.com). Based on the results, the isolates were assigned to both a genus and a species in most cases, except for one strain where identification could not be determined. Due to the limitations of phenotypic methods species identification was confirmed by 16S rRNA sequencing.

#### Identification based on analysis of the 16S rRNA fragment

LAB isolates were identified using 16S rRNA sequence analysis. Genomic DNA was isolated with the Genomic Mini AX Bacteria Spin kit (A&A BIOTECHNOLOGY, Gdańsk, Poland), designed for Gram-positive bacteria, according to the manufacturer’s instructions. The PCR reaction was performed with the Phusion High-Fidelity DNA Polymerase reagent kit (Thermo Fisher Scientific, Waltham, Massachusetts, USA) and the fD1/rP2 primer set (Sigma-Aldrich, Darmstadt, Germany). Sequencing of the PCR products was performed by Genomed (Warsaw, Poland) using Illumina sequencing. The obtained sequences were compared with the NCBI GenBank database using BLAST software. The nucleotide sequences of the isolated LAB strains *Lactiplantibacillus plantarum* P1, P3, P24, P25, P27, P34, *Levilactobacillus brevis* P16, P17, P30, and *Lactiplantibacillus pentosus* P18 were deposited in the GenBank National Centre for Biotechnology Information database under the accession numbers: PP733398, PP733399, PP733406, PP733407, PP733408, PP733410, PP733402, PP733403, PP733409, and PP733404.

### Basic characterization of LAB

#### Presence of catalase

The presence of catalase was tested using the slide method. A loop of bacterial biomass from a 24-hour culture on MRS Agar (Merck) was placed on a glass slide, forming an oval smear about 4 mm in diameter. A 500 µL aliquot of 3% (*w/v*) hydrogen peroxide (Sigma-Aldrich, Darmstadt, Germany) was applied to the biomass. The presence of catalase was confirmed by the appearance of oxygen bubbles. In the absence of catalase, no bubbles were observed.

#### Coagulase

The ability of the isolated strains of LAB to synthesize coagulase was assessed using the tube method. After 4 h of incubation, the culture was observed for clot formation. In line with the assay’s assumption (to confirm the strains were truly negative for free coagulase), the incubation time was extended to 24 h. After this period, the presence or absence of clot formation was re-evaluated. A positive reference sample was a 24-hour culture of *Staphylococcus aureus* ATCC 25923.

#### Hemolytic activity

Hemolytic activity was determined using MRS Agar (Merck) with 5% (*v/v*) defibrinated horse blood (Biomaxima, Lublin, Poland). The plates were incubated at 30 °C for 48 h to observe hemolysis around the colonies. α-hemolysis was identified by a green zones, while β-hemolysis showed a clear zone around the bacterial colonies. γ-hemolysis was characterized by the absence of both green coloration and clear zones^18^.

#### Hydrogen peroxide production

The ability of LAB to produce hydrogen peroxide (H_2_O_2_) was determined using a plate method with a chromogenic compound. A solution of 25 mg 3,3’,5,5’-tetramethylbenzidine (TMB) (Sigma-Aldrich, Darmstadt, Germany) and 1 mg peroxidase (Sigma-Aldrich, Darmstadt, Germany) was added to 100 mL MRS Agar (Merck)^19^. The medium was poured into plates, and after solidification, LAB were inoculated by surface plating. The plates were incubated at 37 °C with 5% (*v/v*) CO_2_ (S@fegrow 188 PRO, EUROCLONE S.p.A, Pero, Italy) for 48 h. After incubation, the color change and the intensity of the violet coloration were evaluated macroscopically. The absence of any color change in the LAB biomass indicated no production of H₂O₂ and was assigned a value of 0. A partial violet coloration of the biomass signified weak H₂O₂ production and corresponded to a value of 1. Complete dark violet coloration of the biomass demonstrated a strong capacity for H₂O₂ synthesis, which was assigned a value of 2.

### Measurement of the enzymatic activity

The enzymatic activity of LAB isolates was evaluated using the API^®^ ZYM (BioMérieux, Marcy l’Etoile, France). Wells were filled with 65 µL of LAB culture and incubated at 30 °C for 4 h. Following the manufacturer’s protocol, ZYM A and ZYM B reagents were added, and color development was observed for 5 min. The strips were then exposed to a strong light source for 10 s. Enzymatic activity was evaluated according to the API^®^ ZYM scale provided by the manufacturer, ranging from 0 (no activity) to 5.

### Phenotypic profile of antibiotic resistance

Antibiotic resistance of the LAB isolates was assessed using the disk diffusion method following EFSA guidelines^20^. 100 µL of a 24-hour LAB culture at a concentration of 1.5 × 10⁸ CFU/mL (0.5 McFarland standard) was spread on the surface of MRS Agar (Merck) plates. Oxoid antibiotic discs (Fisher Scientific International, Hampton, NH, USA) containing ampicillin (2 µg), chloramphenicol (30 µg), gentamicin (10 µg), clindamycin (2 µg), erythromycin (5 µg), kanamycin (30 µg), streptomycin (25 µg), tetracycline (30 µg), and vancomycin (30 µg) were placed on the plates. The plates were incubated at 30 °C for 24 h, and inhibition zones were measured (mean of 3 replicates ± standard deviation). Results were interpreted according to Charteris et al.^21^ and Table S1, classifying the strains as resistant, moderately susceptible, or sensitive based on the range of inhibition zones.

### Mucin degradation

The ability of LAB to hydrolyze mucin was assessed using a spectrophotometric method, following Tarracchini et al.^22^ with modifications. MRS medium without glucose (BTL, Łódź, Poland) was supplemented with 1% (*w/v*) mucin (Sigma-Aldrich, Darmstadt, Germany). In each well of a 96-well plate, 135 µL of medium were added with 15 µL of overnight LAB culture. Positive controls included glucose-free MRS medium without mucin and standard MRS Broth (Merck) containing all components (including glucose), while negative controls used media without LAB. Absorbance at 600 nm was measured at the start and after 48 h of incubation at 30 °C using a CE 2041 UV/VIS Spectrophotometer (Buck Scientific Inc., Norwalk, CT, USA). Results were expressed as OD_600_ ± standard deviation from three independent experiments.

### Biofilm formation coefficient

The ability of LAB to form biofilm was assessed on two surfaces: a biotic surface (polystyrene plate coated with porcine mucin) and an abiotic surface (sterile polystyrene plate). For the biotic surface, 200 µL of 1 mg/mL mucin solution (Sigma-Aldrich) was added to each well of a 96-well plate, incubated at room temperature for 6 h with shaking (100 rpm), and stored for 18 h at 4 °C. Wells were washed three times with sterile phosphate buffered saline (PBS) (pH 7.0). Then, 180 µL of MRS medium (Merck) with 20 µL of 24-hour LAB culture (10⁸–10⁹ CFU/mL) was added to each well. The plates were incubated for 24 h at 30 °C. After incubation, the medium was removed, wells were washed three times with PBS, and dried. Then, 150 µL of 0.05% crystal violet solution was added and plates were incubated for 45 min with shaking. The walls were washed three times with PBS, and crystal violet was extracted with 200 µL of 96% ethanol per well, and transferred to a new sterile 96-well plates. The absorbance of the extracted dye was measured at 490 nm^23^. The biofilm formation coefficient (BFC) was calculated using the formula:

1 $$BFC = \frac{{A~\left( {test} \right)}}{{A~\left( {control} \right)}}$$

BFC - biofilm formation coefficient

A (test) - absorbance of the tested sample

A (control) - absorbance of the control (wells without bacterial inoculation)

### Stress tolerance of LAB isolates

#### LAB growth and tolerance to H_2_O_2_, phenol, sodium chloride

The growth of LAB strains and their survival under adverse environmental conditions, including the presence of H₂O₂, phenol, and high concentrations of sodium chloride (NaCl), were assessed using the turbidimetric method^24–27^ with a microplate reader (Multiskan SkyHigh Microplate Spectrophotometer, Thermo Fisher Scientific, Waltham, MA, USA). MRS Broth (Merck) was used either without additives as a control or supplemented with H₂O₂ (Merck, Darmstadt, Germany), phenol (Merck, Darmstadt, Germany) at 0.4% (*v/v*), or NaCl (Sigma-Aldrich) at 10% (*w/v*). Aliquots of 180 µL of the prepared medium were dispensed into the wells of a 96-well plate, followed by the addition of 20 µL of inoculum from a 24-hour bacterial culture at a concentration of approximately 10⁸ CFU/mL. Samples were incubated at 30 °C, and absorbance was measured at 560 nm at 0, 2, 4, 6, 8, 10, 12, 24, 48, and 72 h of incubation. All samples were analyzed in triplicate.

#### Survival of LAB in the presence of bile salts

The impact of bile salts on the survival of LAB was determined using the plate method. LAB strains at a density of 10^7^ CFU/mL were cultured in MRS Broth (Merck) with bile salts (BTL, Łódź, Poland) at a concentration of 0.4% (*w/v*). Serial dilutions were spread onto MRS Agar (Merck) plates at time intervals of 0, 1, 2, and 3 h during incubation at 30 °C. The results are presented as the mean values of three independent trials, expressed as log colony-forming units (CFU) per milliliter.

#### Survival of LAB at a low pH

The ability of LAB to survive at different pH levels (1.5; 2.0; 2.5; 3.0) was tested using the plate method with MRS Broth (Merck), adjusting the pH with 1 N hydrochloric acid (HCl). The samples were incubated at 30 °C. At specified intervals (0, 1, 2, and 3 h), plate counts on MRS Agar (Merck) were carried out followed by incubation at 30 °C for 48 h. The effect of low pH on LAB survival was expressed as log CFU/mL.

#### Acidification potential of LAB

Determination of the total titratable acidity produced by tested LAB was performed by titration samples after 72 h of incubation at 30 °C, with a 0.1-N aqueous solution of NaOH, in the presence of phenolphthalein. The total titratable acidity is expressed as mL of 0.1-M NaOH used to titrate 1 mL of sample.

### Antimicrobial activity

The antimicrobial activity of LAB was assessed against selected foodborne pathogens, including *Staphylococcus aureus* (ATCC 27734, ATCC 25923, ATCC 29734), *Listeria monocytogene*s (ATCC 7644, ATCC 15313, ATCC 19115, ATCC 19111, ATCC 35152, ATCC 19112), *Escherichia coli* (ATCC 35218, ATCC 11303), *Salmonella* Choleraesuis ATCC 7001, *Salmonella* Enteritidis ATCC 13076, *Salmonella* Typhimurium ATCC 14028. To examine antimicrobial activity using bar method^28^, 100 µL of test bacteria at a concentration of 1.5 × 10⁸ cells/mL (0.5 McFarland standard) was spread on Mueller-Hinton Agar (Merck, Darmstadt, Germany). Bars (0.8 mm diameter) cut from the MRS Agar (Merck) overgrown with LAB (48-hour culture, 30 °C) were placed on the Mueller-Hinton Agar (Merck) with test bacteria. Plates were incubated at 30 °C for *Listeria* spp. and at 37 °C for other strains for 18–24 h. Growth inhibition zones were measured in mm.

### Statistical analysis

Statistical analysis was conducted using StatSoft Statistica program version STATISTICA 10. The experiments were performed in 3 independent repetitions. The results were subjected to one-way Anova statistical analysis, *p* ≤ 0.05, *post hoc* Tukey test. The correlations between average antimicrobial activity and metabolites (H_2_O_2_ and acidifying activity) were estimated based on the Pearson correlation coefficient.

## Results

### Identification of isolated LAB strains

The species affiliation of the characterized LAB strains is presented in Table 1. A convergence of identification between the 16S rRNA method and API 50 CHL fermentation profile tests was observed for strains P1, P3, P24, P25, P27, and P34, where both methods identified them as belonging to the species *Lb. plantarum*, as well as for strain P18, which was confirmed to belong to the species *Lb. pentosus*. However, for strains P16, P17, and P30, molecular biology methods assigned them to the species *Lb. brevis* with a very high identification agreement ranging from 99.91 to 100.00%. In the case of these strains, no correlation was observed between the identification results obtained from molecular methods and API 50 CHL fermentation tests.

**Table 1:** Genotypic and phenotypic identification of isolated LAB cultures.

Strain	16S rRNA		API 50 CHL	
	Identified species	% ID	Identified species	% ID
P1	*Lactiplantibacillus plantarum*	99.92	*Lactiplantibacillus plantarum*	99.90
P3	*Lactiplantibacillus plantarum*	100.00	*Lactiplantibacillus plantarum*	99.60
P16	*Levilactobacillus brevis*	99.92	*Lactiplantibacillus pentosus*	87.70
P17	*Levilactobacillus brevis*	99.91	*Lactiplantibacillus pentosus*	82.90
P18	*Lactiplantibacillus pentosus*	100.00	*Lactiplantibacillus pentosus*	96.00
P24	*Lactiplantibacillus plantarum*	100.00	*Lactiplantibacillus plantarum*	99.10
P25	*Lactiplantibacillus plantarum*	99.60	*Lactiplantibacillus plantarum*	99.90
P27	*Lactiplantibacillus plantarum*	99.76	*Lactiplantibacillus plantarum*	99.90
P30	*Levilactobacillus brevis*	100.00	*Lactiplantibacillus plantarum*	Profile unacceptable
P34	*Lactiplantibacillus plantarum*	100.00	*Lactiplantibacillus plantarum*	99.90

### Characterization of LAB

**Fig. 1:**
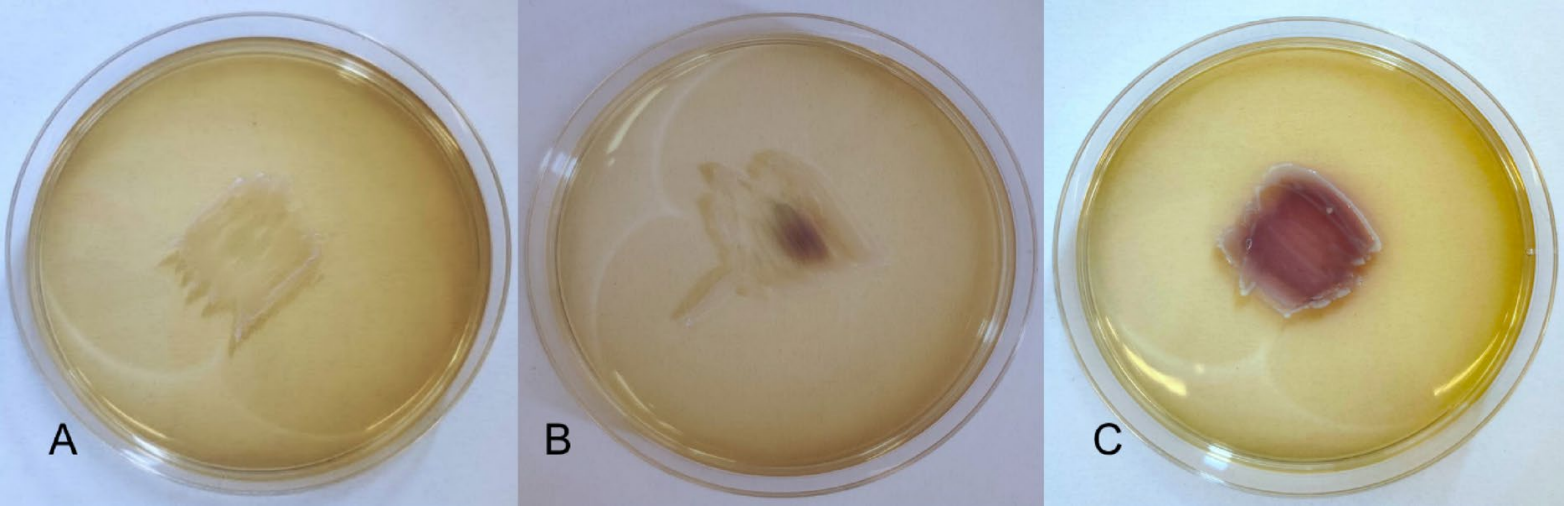
Visualization of the qualitative determination of the ability to synthesize H₂O₂ by LAB in the presence of the TMB chromogen. (**A**) growth of the *Lb. brevis* P17 (no H_2_O_2_ production - value 0); (**B**) growth of the *Lb. pentosus* P18 (weak H_2_O_2_ production - value 1); (**C**) growth of the *Lb. plantarum* P27 (strong ability to synthesize H_2_O_2_ - value 2).

The characterization of LAB strains isolated from onions is delineated in Table S2. The tested strains differed slightly in their appearance under the microscope. However, all of them were catalase-negative, coagulase-negative, classified as Gram-positive bacilli, and did not show any hemolytic activity, indicating γ-hemolysis. Moreover, *Lb. brevis* P16, P17, and P30 did not produce H_2_O_2_, as evidenced by the absence of color change in the biomass (Fig. 1A). In contrast, five strains (*Lb. plantarum* P1, P3, P24, P34, and *Lb. pentosus* P18) showed weak H_2_O_2_ synthesis, resulting in partial coloration of the biomass (Fig. 1B). The most effective H_2_O_2_ producers were *Lb. plantarum* P25 and P27, with their entire biomass turning dark violet on solid medium with TMB chromogen (Fig. 1C).

### Carbohydrate metabolism

All tested LAB exhibited the ability to ferment several sugars, including D-ribose, D-glucose, D-fructose, D-mannose, D-mannitol, arbutin, esculin, salicine, D-cellobiose, D-maltose, D-saccharose, D-melezitose, and D-turanose. Conversely, none of the strains fermented compounds such as erythritol, D-arabinose, L-xylose, amidon, glycogen, xylitol, D-adonitol, methyl-D-xylopyranoside, dulcitol, L-sorbose, inositol, D-lyxose, D-tagatose, D-fucose, L-fucose, D-arabitol, L-arabitol, 2-keto-gluconate, or 5-keto-gluconate (Table 2). Metabolic variability was evident among the strains for less common substrates. *Lb. pentosus* P18 and *Lb. plantarum* P25 were the only strains unable to ferment gentiobiose. Fermentation of L-arabinose was observed in most strains except for *Lb. plantarum* P3 and *Lb. brevis* P30, suggesting a limited capacity of these strains to utilize this substrate. Rare metabolic capabilities were noted for specific strains. Glycerol fermentation was observed exclusively in *Lb. brevis* P16 and *Lb. plantarum* P24. Potassium gluconate fermentation was limited to *Lb. plantarum* P1, P3, and P25. Fermentation of D-xylose was detected in six strains (*Lb. plantarum* P3, P24, *Lb. brevis* P16, P17, P30, *Lb. pentosus* P18), while L-rhamnose was fermented by four strains (*Lb. plantarum* P3, P24, P27, *Lb. brevis* P16). Additionally, inulin fermentation was limited to five strains (*Lb. plantarum* P1, P3, P24, *Lb. brevis* P16, P17).

**Table 2:** Metabolic activity of selected LAB strains.

Type of test		Lactic acid bacteria									
		P1	P3	P16	P17	P18	P24	P25	P27	P30	P34
control	CTRL	-	-	-	-	-	-	-	-	-	-
glycerol	GLY	-	-	+	-	-	+	-	-	-	-
erythritol	ERY	-	-	-	-	-	-	-	-	-	-
D-arabinose	DARA	-	-	-	-	-	-	-	-	-	-
L-arabinose	LARA	+	-	+	+	+	+	+	+	-	+
D-ribose	RIB	+	+	+	+	+	+	+	+	+	+
D-xylose	DXYL	-	+	+	+	+	+	-	-	+	-
L-xylose	LXYL	-	-	-	-	-	-	-	-	-	-
D-adonitol	ADO	-	-	-	-	-	-	-	-	-	-
methyl-ß-D-xylopyranoside	MDX	-	-	-	-	-	-	-	-	-	-
D-galactose	GAL	+	+	+	-	+	+	+	+	+	+
D-glucose	GLU	+	+	+	+	+	+	+	+	+	+
D-fructose	FRU	+	+	+	+	+	+	+	+	+	+
D-mannose	MNE	+	+	+	+	+	+	+	+	+	+
L-sorbose	SBE	-	-	-	-	-	-	-	-	-	-
L-rhamnose	RHA	-	+	+	-	-	+	-	+	-	-
dulcitol	DUL	-	-	-	-	-	-	-	-	-	-
inositol	INO	-	-	-	-	-	-	-	-	-	-
D-mannitol	MAN	+	+	+	+	+	+	+	+	+	+
D-sorbitol	SOR	+	+	+	-	+	+	+	+	+	+
methyl-D-mannopyranoside	MDM	+	+	+	-	-	+	+	+	+	+
methyl-D-glucopyranoside	MDG	-	+	+	-	-	+	-	+	+	-
N-acetylglucosamine	NAG	+	+	+	-	+	+	+	+	+	+
amygdalin	AMY	+	+	+	+	+	+	+	+	-	+
arbutin	ARB	+	+	+	+	+	+	+	+	+	+
esculin	ESC	+	+	+	+	+	+	+	+	+	+
salicin	SAL	+	+	+	+	+	+	+	+	+	+
D-cellobiose	CEL	+	+	+	+	+	+	+	+	+	+
D-maltose	MAL	+	+	+	+	+	+	+	+	+	+
D-lactose	LAC	+	+	+	-	+	+	+	+	+	+
D-melibiose	MEL	+	+	+	+	+	+	+	+	-	+
sucrose	SAC	+	+	+	+	+	+	+	+	+	+
D-trehalose	TRE	+	+	+	+	+	+	+	+	-	+
inulin	INU	+	+	+	+	-	+	-	-	-	-
D-melezitose	MLZ	+	+	+	+	+	+	+	+	+	+
D-raffinose	RAF	+	+	+	-	+	+	+	+	+	+
starch	AMD	-	-	-	-	-	-	-	-	-	-
glycogen	GLYG	-	-	-	-	-	-	-	-	-	-
xylitol	XLT	-	-	-	-	-	-	-	-	-	-
gentiobiose	GEN	+	+	+	+	-	+	-	+	+	+
D-turanose	TUR	+	+	+	+	+	+	+	+	+	+
D-lyxose	LYX	-	-	-	-	-	-	-	-	-	-
D-tagatose	TAG	-	-	-	-	-	-	-	-	-	-
D-fucose	DFUC	-	-	-	-	-	-	-	-	-	-
L-fucose	LFUC	-	-	-	-	-	-	-	-	-	-
D-arabitol	DARL	-	-	-	-	-	-	-	-	-	-
L-arabitol	LARL	-	-	-	-	-	-	-	-	-	-
potassium gluconate	GNT	+	+	-	-	-	-	+	-	-	-
2-keto-gluconate	2KG	-	-	-	-	-	-	-	-	-	-
5-keto-gluconate	5KG	-	-	-	-	-	-	-	-	-	-

### Enzymatic activity

**Fig. 2:**
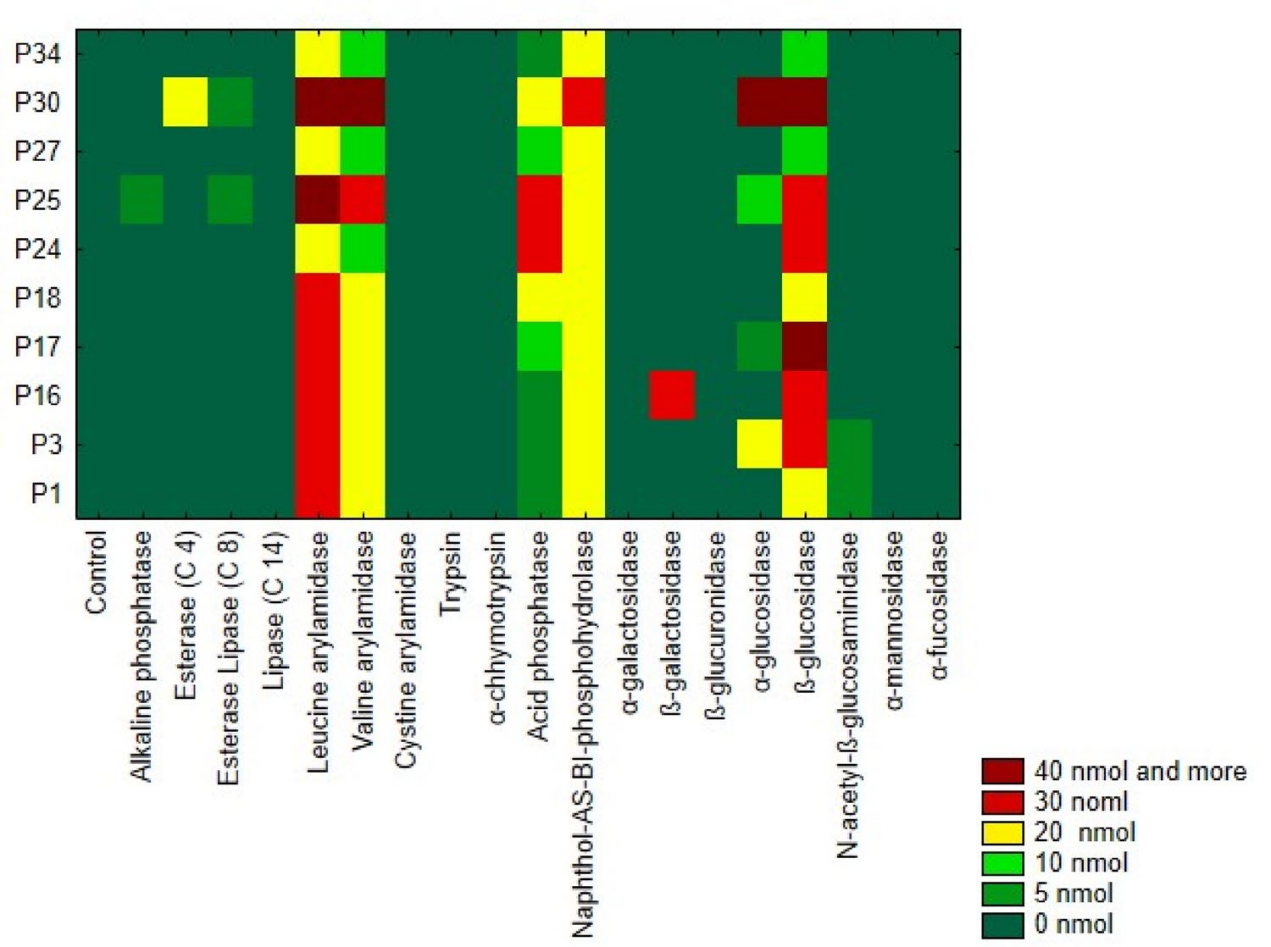
Enzymatic activity of LAB isolated from onions (*Allium cepa* L.). P1-P34 LAB strains described in Table 1. The color scale represents enzymatic activity levels, ranging from green (0 nmol) to red (40 nmol and above).

The enzymatic activity of LAB strains is reported in Fig. 2. All strains exhibited activity for Naphthol-AS-BI-phosphohydrolase and β-glucosidase, with most strains showing moderate to high activity levels (20–40 nmol). *Lb. plantarum* P30 demonstrated notably high activity for both enzymes, reaching up to 30–40 nmol. Additionally, all strains showed activity for leucine arylamidase, with the highest levels observed in *Lb. plantarum* P25 and *Lb. brevis* P30 (40 nmol). Valine arylamidase activity varied across strains, with the lowest activity in *Lb. plantarum* P24, P27, and P34, and the highest in *Lb. plantarum* P25 and *Lb. brevis* P30. Acid phosphatase activity ranged from 5 to 30 nmol, with *Lb. plantarum* P24 and P25 showing the highest levels. Selective enzymatic activity was observed for α-glucosidase, with only *Lb. plantarum* P3 and *Lb. brevis* P30 demonstrating significant activity. N-acetyl-β-glucosaminidase was synthesized by *Lb. plantarum* P1 and P3 at low levels (5 nmol), while β-galactosidase activity was exclusive to *Lb. brevis* P16. None of the strains displayed activity for lipase (C14), cystine arylamidase, trypsin, α-galactosidase, β-glucuronidase, α-mannosidase, or α-fucosidase, indicating limited enzymatic capability in metabolizing these substrates.

### Phenotypic profile of antibiotic resistance of LAB

The antibiotics used in this study were classified into two groups based on their mechanisms of action against bacteria (Table S1). For the first group, all tested strains of LAB exhibited no zones of growth inhibition for vancomycin, thus categorizing them as resistant to this antibiotic. In contrast, the inhibition zones for ampicillin varied among the LAB strains, ranging from 13.75 ± 0.50 mm to 26.75 ± 0.96 mm (Table S3). Based on the criteria presented in Table S1, two LAB strains were classified as moderately susceptible (MS) to ampicillin, while eight strains were identified as susceptible (S) (Table 3).

Regarding the second group of antibiotics, all tested LAB strains demonstrated complete resistance (100%) to streptomycin, kanamycin, and gentamicin. For clindamycin, three strains were categorized as resistant, and seven strains as moderately susceptible. Notably, all tested LAB strains were sensitive to tetracycline, chloramphenicol, and erythromycin.

**Table 3:** In vitro activity selected antibiotics towards tested LAB.

Antimicrobial agent Name (Abbreviation) Concentration (µg)	R (%)	MS (%)	S (%)
Vancomycin (VA) 30	10 (100%)	-	-
Ampicilin (AMP) 2	-	2 (20%)	8 (80%)
Tetracycline (TE) 30	-	-	10 (100%)
Streptomycin (S) 25	10 (100%)	-	-
Kanamycin (K) 30	10 (100%)	-	-
Gentamicin (CN) 10	10 (100%)	-	-
Chloramphenicol (C) 30	-	-	10 (100%)
Erythromycin (E) 5	-	-	10 (100%)
Clindamycin (DA) 2	3 (30%)	7 (70%)	-

R- resistant; MS - moderately susceptible; S – sensitive; 100% − 10 strains of LAB.

### Mucin degradation ability of LAB

**Fig. 3:**
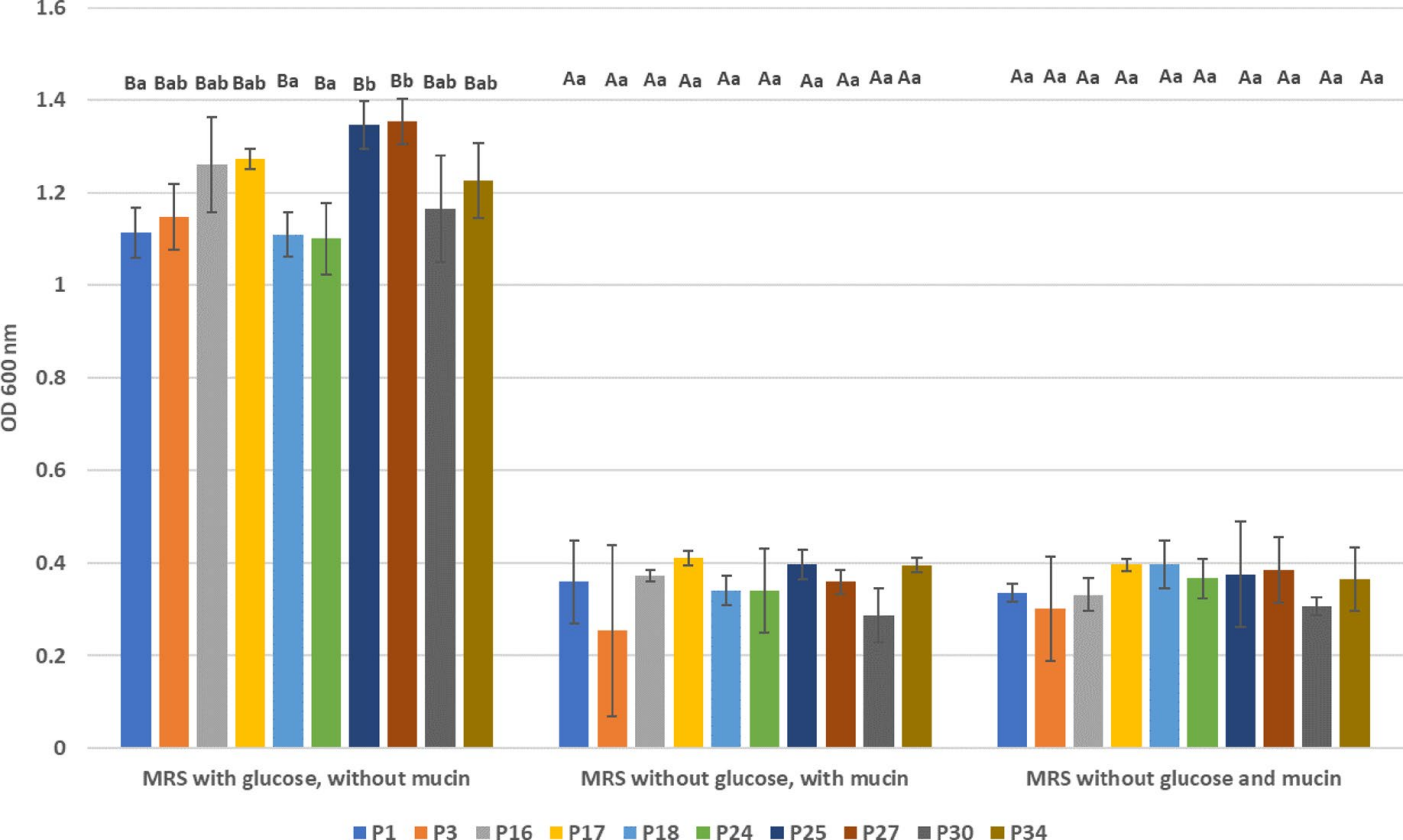
The growth of the tested strains of LAB in the presence of mucin as a carbon source. a, b – statistical differences between LAB strains within the same medium (ANOVA, *post-hoc* Tukey test, *p* ≤ 0.05); A, B – statistical differences within the same strain between media (ANOVA, *post-hoc* Tukey test, *p* ≤ 0.05); P1-P34 LAB strains described in Table 1.

Figure 3 presents the growth of isolated LAB strains in the presence of mucin as a carbon source. In the control condition with MRS medium containing glucose but lacking mucin, all strains exhibited significant growth, with optical densities (OD_600_) ranging from 1.11 to 1.35. Conversely, strains cultured in MRS medium with mucin but without glucose showed markedly reduced growth, with optical densities ranging from 0.25 to 0.41. A second control, consisting of MRS medium without both mucin and glucose, resulted in optical densities ranging from 0.30 to 0.39. The results indicate that the isolated LAB strains are unable to metabolize mucin.

### Ability of LAB to form biofilm

Table 4 presents the results of the analysis of the ability of LAB to form biofilms on abiotic and biotic surfaces. The BFC values of LAB isolated from onions on the abiotic surface (polystyrene plate) ranged from 1.37 to 5.47. Statistically, the highest ability to form a biofilm on the abiotic surface was observed in *Lb. plantarum* P24, which showed a significantly higher BFC compared to the other tested strains. In the remaining strains, the BFC values were lower, not exceeding 3.5. On the biotic surface (polystyrene plate coated with mucin), the BFC values ranged from 0.65 to 1.54. The highest BFC value was observed in *Lb. plantarum* P1, and the lowest in *Lb. plantarum* P34.

**Table 4:** Biofilm formation factor of the tested LAB.

LAB strain	Biofilm formation coefficient BFC	
	Abiotic surface	Biotic surface
P1	3.33 ± 0.72 ^Bb^	1.54 ± 0.30 ^Ab^
P3	3.15 ± 0.33 ^Bab^	0.81 ± 0.39 ^Aa^
P16	2.08 ± 0.48 ^Bab^	0.97 ± 0.27 ^Aab^
P17	2.56 ± 0.39 ^Bab^	1.17 ± 0.37 ^Aab^
P18	2.81 ± 0.14 ^Bab^	1.05 ± 0.13 ^Aab^
P24	5.47 ± 1.65 ^Bc^	1.09 ± 0.17 ^Aab^
P25	1.56 ± 0.17 ^Bab^	0.72 ± 0.18 ^Aa^
P27	2.34 ± 0.22 ^Bab^	0.71 ± 0.20 ^Aa^
P30	1.53 ± 0.40 ^Aab^	1.03 ± 0.12 ^Aab^
P34	1.37 ± 0.08 ^Ba^	0.65 ± 0.14 ^Aa^

A, B – statistical differences (ANOVA, Tukey’s *post-hoc* test (*p* ≤ 0.05)) between abiotic surface and biotic surface; a, b, c – statistical differences (ANOVA, Tukey’s *post-hoc* test (*p* ≤ 0.05)) between strains on the same surface.

In 9 out of the 10 tested strains, the BFC value was statistically significantly higher on the abiotic surface than on the biotic surface. The only exception was *Lb. brevis* P30, where the BFC value on the abiotic surface was higher; however this difference was not statistically significant when compared to the BFC value on the biotic surface.

### Survival of LAB in adverse environmental conditions

#### The growth of LAB in the presence of H_2_O_2_, phenol, and sodium chloride

**Fig. 4:**
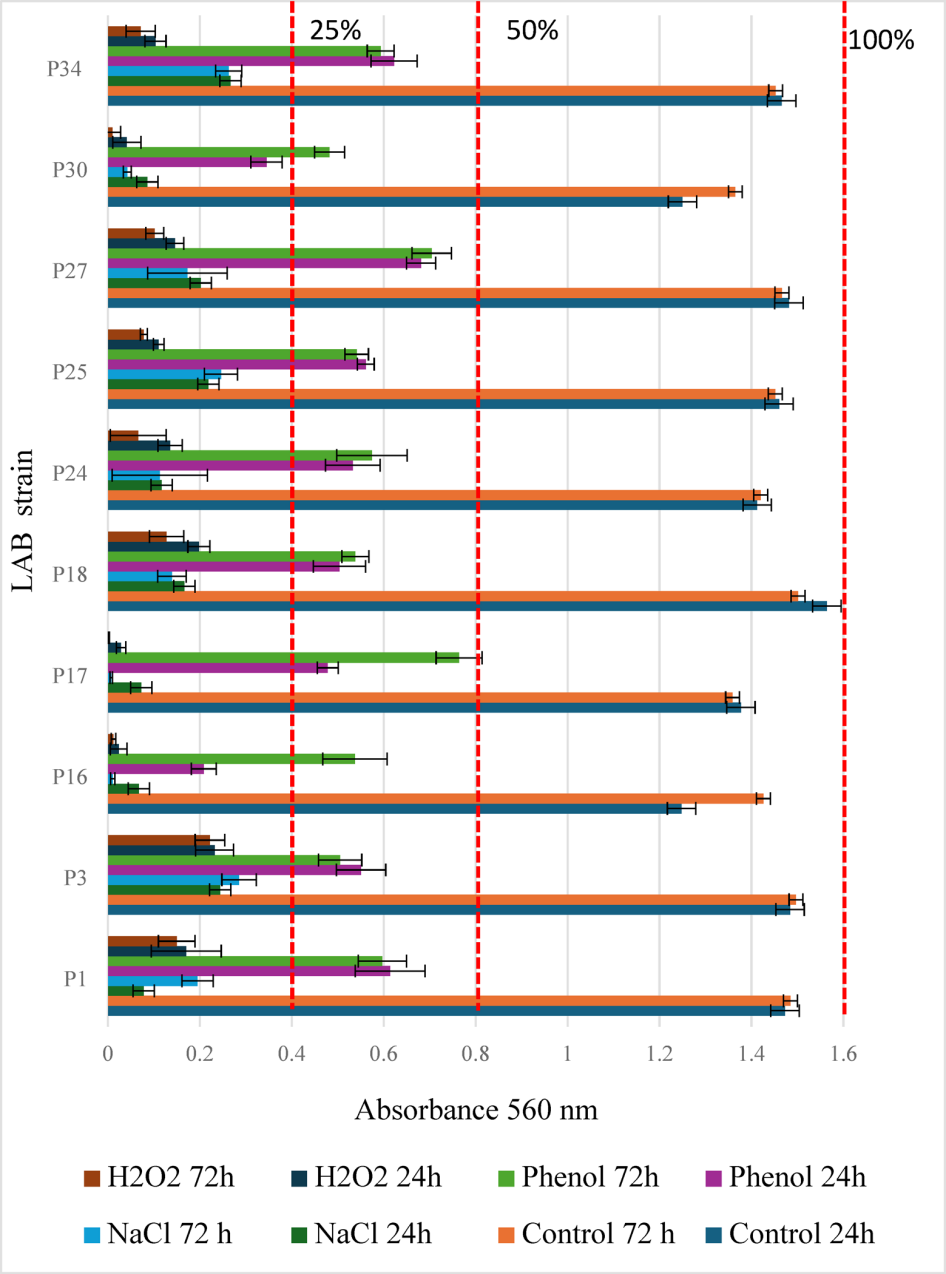
Growth of LAB in the presence of adverse environmental factors after 24 and 72 h.

The growth of bacteria in the presence of environmental stress factors was assessed at 0, 2, 4, 6, 8, 10, 12, 24, 48, and 72 h (Fig. S2). However, to capture the most significant differences between strains, the results for 24 h (the end of the logarithmic growth phase) and 72 h (the bacterial death phase) are presented in Fig. 4. It is worth noting that *Lb. brevis* strains exhibited slower biomass growth during the 12-hour incubation compared to the other tested strains. Moreover, for *Lb. brevis* P16 and P30, the increase in biomass after 24 h was significantly lower than after 72 h (Table 5). Only *Lb. pentosus* P18 showed a statistically significant decrease in viable cell count (log CFU/mL) after 72 h compared to 24 h.

All stress factors (NaCl, H₂O₂, phenol) decreased growth intensity compared to the control samples (statistically significant differences, Table 5). Strains P16, P17, and P30, belonging to the species *Lb. brevis*, exhibited higher sensitivity to these adverse factors compared to strains assigned to *Lb. plantarum* (P1, P3, P24, P25, P27, P34) and *Lb. pentosus* (P18). All the tested LAB strains displayed varying levels of tolerance in the presence of phenol in the growth environment. *Lb. brevis* demonstrated a statistically significant increase in absorbance after 72 h compared to 24 h.

**Table 5:** Statistical analysis for the values presented in Fig. 4.

Time	Sample	LAB strain									
		P1	P3	P16	P17	P18	P24	P25	P27	P30	P34
24 h	Control	D	C	F	E	D	C	D	D	D	D
	NaCl	A	A	B	B	A	A	B	B	A	B
	Phenol	C	B	C	C	B	B	C	C	B	C
	H_2_**O**_**2**_	B	A	AB	B	A	A	A	A	A	A
72 h	Control	D	C	E	E	C	C	D	D	E	D
	NaCl	AB	A	A	A	A	A	B	AB	A	B
	Phenol	C	B	D	D	B	B	C	C	C	C
	H_2_**O**_**2**_	B	A	A	A	A	A	A	A	A	A

A, B, C, D – statistical differences within the same strain (ANOVA, *post-hoc* Tukey test, *p* ≤ 0.05).

#### Bile salts and low pH

The results showed that all tested LAB demonstrated high survivability in 0.4% (*w/v*) bile salts, with viable counts ranging from 6.84 to 7.46 log CFU/mL after 3 h of incubation (Table 6), compared with 7.03 to 7.75 log CFU/mL in the control samples. Moreover, the examined LAB displayed roughly similar viable count responses upon incubation at pH 3.0 (10^7^ CFU/mL after 4 h of incubation for all tested strains). However, their survival rates declined significantly (*p* < 0.05) at pH < 3.0, particularly after 2 h of incubation. Specifically, only *Lb. plantarum* P27 did not exhibit a statistically significant reduction in bacterial count after one hour of incubation at pH 2.5 compared to the control, although prolonged exposure eventually led to a noticeable decline in its viability. After 4 h of incubation at pH 2.5, the highest bacterial counts were observed for *Lb. brevis* P17 (4.35 log CFU/mL) and P30 (4.09 log CFU/mL), whereas *Lb. plantarum* P27 and P34 were not detected. At pH 2.0 only *Lb. plantarum* P3 (4.91 log CFU/mL) was observed after one hour of incubation at 30 °C, however, by the second hour of incubation, no presence of these bacteria was noted. At a pH of 1.5, none of the tested LAB were detectable after just one hour of incubation.

**Table 6:** Number of LAB in the presence of bile salts and at low pH.

Stress factor	Time [h]	LAB strain									
		P1	P3	P16	P17	P18	P24	P25	P27	P30	P34
Control	0	7.78 ± 0.12 ^f^	7.70 ± 0.04 ^f^	7.02 ± 0.02 ^e^	7.28 ± 0.13 ^c^	7.78 ± 0.07 ^f^	7.63 ± 0.06 ^f^	7.63 ± 0.13 ^f^	7.51 ± 0.09 ^c^	7.17 ± 0.13 ^e^	7.64 ± 0.14 ^d^
	1	7.72 ± 0.05 ^f^	7.71 ± 0.16 ^f^	7.00 ± 0.07 ^e^	7.24 ± 0.11 ^c^	7.73 ± 0.10 ^f^	7.64 ± 0.04 ^f^	7.42 ± 0.27 e^f^	7.53 ± 0.07 ^c^	7.00 ± 0.04 ^e^	7.61 ± 0.01 ^d^
	2	7.72 ± 0.04 ^f^	7.71 ± 0.06 ^f^	7.00 ± 0.09 ^e^	7.21 ± 0.16 ^c^	7.71 ± 0.09 ^f^	7.68 ± 0.05 ^f^	7.60 ± 0.05 ^f^	7.53 ± 0.07 ^c^	7.04 ± 0.06 ^e^	7.66 ± 0.14 ^d^
	3	7.75 ± 0.09 ^f^	7.69 ± 0.12 ^ef^	7.03 ± 0.10 ^e^	7.11 ± 0.12 ^c^	7.70 ± 0.11 ^f^	7.72 ± 0.11 ^f^	7.77 ± 0.06 ^f^	7.60 ± 0.08 ^c^	7.06 ± 0.08 ^e^	7.66 ± 0.10 ^d^
Bile salts	0	7.78 ± 0.12 ^f^	7.70 ± 0.04 ^ef^	7.02 ± 0.02 ^e^	7.28 ± 0.13 ^c^	7.78 ± 0.07 ^f^	7.63 ± 0.06 ^f^	7.63 ± 0.13 ^e^	7.51 ± 0.09 ^c^	7.17 ± 0.13 ^e^	7.64 ± 0.14 ^d^
	1	7.56 ± 0.13 ^e^	7.61 ± 0.09 ^e^	6.96 ± 0.05 ^e^	7.08 ± 0.08 ^c^	7.50 ± 0.06 ^e^	7.50 ± 0.07 ^f^	7.29 ± 0.26 ^e^	7.52 ± 0.17 ^c^	7.28 ± 0.19 ^e^	7.49 ± 0.13 ^d^
	2	7.52 ± 0.19 ^e^	7.52 ± 0.11 ^e^	6.91 ± 0.04 ^e^	7.05 ± 0.05 ^c^	7.42 ± 0.05 ^e^	7.22 ± 0.17 ^e^	7.20 ± 0.18 ^e^	7.47 ± 0.15 ^c^	7.17 ± 0.03 ^e^	7.46 ± 0.14 ^d^
	3	7.49 ± 0.19 ^e^	7.48 ± 0.06 ^e^	6.84 ± 0.06 ^e^	7.07 ± 0.16 ^c^	7.29 ± 0.19 ^e^	7.03 ± 0.13 ^e^	7.20 ± 0.13 ^e^	7.43 ± 0.04 ^c^	7.00 ± 0.11 ^e^	7.46 ± 0.19 ^d^
pH 3.0	0	7.63 ± 0.06 ^f^	7.82 ± 0.04 ^f^	7.27 ± 0.06 ^e^	7.25 ± 0.22 ^c^	7.75 ± 0.08 ^f^	7.68 ± 0.07 ^f^	7.53 ± 0.19 ^e^	7.60 ± 0.11 ^c^	7.31 ± 0.32 ^e^	7.32 ± 0.34 ^d^
	1	7.58 ± 0.10 ^e^	7.67 ± 0.07 ^e^	7.19 ± 0.16 ^e^	7.22 ± 0.16 ^c^	7.61 ± 0.01 ^f^	7.57 ± 0.09 ^f^	7.41 ± 0.18 ^e^	7.55 ± 0.12 ^c^	7.25 ± 0.16 ^e^	7.48 ± 0.14 ^d^
	2	7.57 ± 0.03 ^e^	7.61 ± 0.18 ^e^	7.10 ± 0.13 ^e^	7.22 ± 0.20 ^c^	7.60 ± 0.02 ^f^	7.47 ± 0.02 ^f^	7.55 ± 0.15 ^e^	7.37 ± 0.25 ^c^	7.24 ± 0.16 ^e^	7.36 ± 0.18 ^d^
	3	7.56 ± 0.09 ^e^	7.61 ± 0.06 ^e^	7.25 ± 0.20 ^e^	7.14 ± 0.20 ^c^	7.48 ± 0.01 ^e^	7.44 ± 0.22 ^f^	7.52 ± 0.03 ^e^	7.36 ± 0.32 ^c^	7.24 ± 0.04 ^e^	7.32 ± 0.16 ^d^
	4	7.54 ± 0.21 ^e^	7.61 ± 0.12 ^e^	7.20 ± 0.15 ^e^	7.14 ± 0.11 ^c^	7.30 ± 0.07 ^e^	7.45 ± 0.10 ^f^	7.54 ± 0.21 ^e^	7.26 ± 0.24 ^c^	7.12 ± 0.10 ^e^	7.40 ± 0.13 ^d^
pH 2.5	0	7.64 ± 0.20 ^f^	7.66 ± 0.08 ^e^	7.22 ± 0.21 ^e^	7.23 ± 0.25 ^c^	7.70 ± 0.11 ^f^	7.67 ± 0.05 ^f^	7.65 ± 0.05 ^e^	7.66 ± 0.06 ^c^	7.21 ± 0.18 ^e^	7.63 ± 0.06 ^d^
	1	7.06 ± 0.06 ^d^	6.21 ± 0.22 ^d^	5.30 ± 0.06 ^d^	5.14 ± 0.24 ^b^	4.77 ± 0.15 ^d^	5.91 ± 0.19 ^d^	5.06 ± 0.22 ^d^	7.56 ± 0.07 ^c^	5.33 ± 0.04 ^d^	4.54 ± 0.21 ^c^
	2	4.52 ± 0.05 ^c^	4.56 ± 0.08 ^c^	2.87 ± 0.12 ^c^	4.47 ± 0.04 ^a^	3.57 ± 0.16 ^c^	3.84 ± 0.05 ^c^	4.49 ± 0.15 ^c^	2.59 ± 0.30 ^b^	4.79 ± 0.06 ^c^	1.59 ± 0.11 ^b^
	3	2.75 ± 0.06 ^b^	2.50 ± 0.07 ^b^	2.59 ± 0.03 ^b^	4.43 ± 0.16 ^a^	3.20 ± 0.16 ^b^	3.31 ± 0.20 ^b^	4.04 ± 0.06 ^b^	1.22 ± 0.09 ^a^	4.31 ± 0.03 ^b^	0.88 ± 0.09 ^a^
	4	1.48 ± 0.05 ^a^	0.36 ± 0.39 ^a^	2.31 ± 0.05 ^a^	4.35 ± 0.32 ^a^	2.73 ± 0.02 ^a^	2.66 ± 0.33 ^a^	3.66 ± 0.30 ^a^	n.d.	4.09 ± 0.09 ^a^	n.d.
pH 2.0	0	7.64 ± 0.20 ^f^	7.66 ± 0.08	7.22 ± 0.21 ^e^	7.23 ± 0.25 ^c^	7.70 ± 0.11 ^f^	7.67 ± 0.05 ^f^	7.65 ± 0.05	7.66 ± 0.06 ^c^	7.21 ± 0.18 ^e^	7.63 ± 0.06 ^d^
	1	n.d.	4.91 ± 0.28 ^c^	n.d.	n.d.	n.d.	n.d.	n.d.	n.d.	n.d.	n.d.
	2	n.d.	n.d.	n.d.	n.d.	n.d.	n.d.	n.d.	n.d.	n.d.	n.d.
	3	n.d.	n.d.	n.d.	n.d.	n.d.	n.d.	n.d.	n.d.	n.d.	n.d.
	4	n.d.	n.d.	n.d.	n.d.	n.d.	n.d.	n.d.	n.d.	n.d.	n.d.

n.d.- the growth not detected; a, b, c, d, e, f – statistical differences within the same strain under various stress factors (ANOVA, *post-hoc* Tukey test, *p* ≤ 0.05).

### Antimicrobial activity of LAB

The antimicrobial activity of isolated LAB against 13 strains of pathogenic bacteria transmitted through food was assessed (Table 7). All tested LAB were capable of inhibiting selected pathogenic strains. However, this effect is strain-specific; for example, *Lb. brevis* P30 did not inhibit the growth of *S. aureus* ATCC 25923, and the *Lb. brevis* P17 did not inhibit the growth of *S. aureus* ATCC 29734.

**Table 7:** Antimicrobial activity of LAB against pathogenic bacteria.

Pathogenic bacteria	LAB inhibition zones ± SD [mm]									
	*Lb. plantarum* P1	*Lb. plantarum* P3	*Lb. brevis* P16	*Lb. brevis* P17	*Lb. pentosus* P18	*Lb. plantarum* P24	*Lb. plantarum* P25	*Lb. plantarum* P27	*Lb. brevis* P30	*Lb. plantarum* P34
*S. aureus* ATCC 27734	20.3 ± 0.50 ^c^	20.5 ± 0.58 ^c^	15.0 ± 2.31 ^c^	6.5 ± 7.51 ^a^	16.8 ± 0.96 ^c^	16.0 ± 0.00 ^c^	18.0 ± 0.00 ^c^	16.5 ± 0.58 ^c^	11.5 ± 0.58 ^b^	17.5 ± 0.58 ^c^
*S. aureus* ATCC 25923	17.8 ± 2.63 ^c^	18.3 ± 2.06 ^c^	5.5 ± 6.35 ^b^	14.0 ± 2.31 ^c^	17.3 ± 0.50 ^c^	17.8 ± 0.50 ^c^	20.3 ± 0.050 ^c^	20.0 ± 0.00 ^c^	0.0 ± 0.00 ^a^	19.5 ± 1.73 ^c^
*S. aureus* ATCC 29734	16.5 ± 1.29 ^b^	19.3 ± 1.50 ^b^	12.8 ± 0.96 ^b^	0.0 ± 0.00 ^a^	18.8 ± 0.96 ^b^	19.3 ± 1.26 ^b^	21.3 ± 1.50 ^b^	20.8 ± 0.50 ^b^	13.5 ± 0.58 ^b^	19.00 ± 0.00 ^b^
*L. monocytogenes* ATCC 7644	23.5 ± 0.58 ^d^	22.0 ± 0.00 ^d^	21.8 ± 0.96 ^d^	21.3 ± 0.50 ^d^	23.00 ± 1.15 ^c^	19.5 ± 0.58 ^b^	22.3 ± 0.50 ^d^	20.3 ± 0.96 ^dc^	16.5 ± 0.58 ^a^	20.5 ± 1.29 ^dc^
*L. monocytogenes* ATCC 15313	21.8 ± 0.50 ^b^	21.0 ± 0.82 ^b^	17.7 ± 0.96 ^a^	19.0 ± 1.15 ^a^	20.8 ± 0.96 ^b^	20.5 ± 0.58 ^b^	20.5 ± 0.58 ^b^	20.5 ± 0.58 ^b^	30.0 ± 5.77 ^c^	31.5 ± 6.35 ^c^
*L. monocytogenes* ATCC 19115	22.8 ± 0.50 ^c^	21.8 ± 0.96 ^c^	18.5 ± 0.58 ^b^	17.8 ± 0.96 ^b^	22.0 ± 1.41 ^c^	23.0 ± 0.00 ^c^	23.0 ± 0.82 ^c^	21.8 ± 0.96 ^c^	16.3 ± 0.50 ^a^	21.8 ± 1.73 ^c^
*L. monocytogenes* ATCC 19111	20.5 ± 0.58 ^a^	23.0 ± 0.82 ^b^	20.3 ± 1.50 ^a^	20.5 ± 1.29 ^a^	23.3 ± 0.50 ^b^	21.3 ± 0.05 ^a^	22.3 ± 0.50 ^a^	23.8 ± 0.96 ^b^	22.0 ± 0.00 ^a^	25.0 ± 0.00 ^c^
*L. monocytogenes* ATCC 35152	28.5 ± 1.00 ^c^	26.0 ± 0.82 ^c^	23.5 ± 1.73 ^a^	22.5 ± 1.73 ^a^	24.5 ± 1.00 ^abc^	25.0 ± 0.00 ^b^	25.0 ± 0.00 ^b^	24.5 ± 1.73 ^abc^	21.0 ± 2.16 ^a^	22.5 ± 0.58 ^a^
*L. monocytogenes* ATCC 19112	22.8 ± 1.71 ^a^	25.3 ± 1.26 ^b^	23.3 ± 2.36 ^a^	22.8 ± 2.87 ^a^	23.25 ± 1.71 ^a^	26.3 ± 2.50 ^b^	23.8 ± 1.26 ^a^	25.8 ± 0.96 ^b^	23.5 ± 2.38 ^a^	25.0 ± 0.82 ^b^
*E. coli* ATCC 35218	16.3 ± 4.43 ^b^	14.5 ± 4.12 ^b^	17.5 ± 0.58 ^b^	18.3 ± 0.96 ^b^	16.5 ± 4.80 ^b^	14.8 ± 4.35 ^b^	15.8 ± 3.86 ^b^	16.0 ± 4.08 ^b^	14.5 ± 0.58 ^b^	13.6 ± 3.35 ^a^
*E. coli* ATCC 11303	16.8 ± 5.50 ^b^	16.5 ± 5.80 ^b^	17.0 ± 0.82 ^b^	17.5 ± 0.58 ^b^	16.5 ± 5.26 ^b^	14.0 ± 4.06 ^a^	17.8 ± 6.08 ^b^	16.3 ± 4.43 ^b^	13.0 ± 0.00 ^a^	13.9 ± 4.21 ^a^
*S.* Choleraesuis ATCC 7001	18.0 ± 1.63 ^d^	16.8 ± 0.96 ^cd^	14.3 ± 0.96 ^c^	13.5 ± 1.00 ^ab^	16.8 ± 0.96 ^cd^	19.25 ± 1.26 ^d^	20.0 ± 0.82 ^d^	19.3 ± 0.96 ^d^	12.8 ± 0.50 ^a^	15.5 ± 0.58 ^c^
*S.* Enteritidis ATCC 13076	15.3 ± 0.96 ^a^	15.5 ± 0.58 ^a^	13.5 ± 1.00 ^a^	14.0 ± 1.15 ^a^	15.5 ± 0.58 ^a^	14.5 ± 0.58 ^a^	15.5 ± 1.29 ^a^	14.3 ± 0.96 ^a^	13.5 ± 1.73 ^a^	16.5 ± 2.38 ^a^
*S.* Typhimurium ATCC 14028	13.8 ± 1.50 ^a^	14.5 ± 1.73 ^a^	14.3 ± 0.50 ^a^	13.8 ± 0.50 ^a^	15.8 ± 0.96 ^a^	13.5 ± 0.58 ^a^	14.8 ± 0.50 ^a^	15.0 ± 1.15 ^a^	12.5 ± 0.58 ^a^	16.0 ± 0.82 ^a^

a, b, c, d–statistical differences, LAB strains were compared among themselves within one test strain (horizontally) (ANOVA, *post-hoc* Tukey test, *p* ≤ 0.05).

**Fig. 5:**
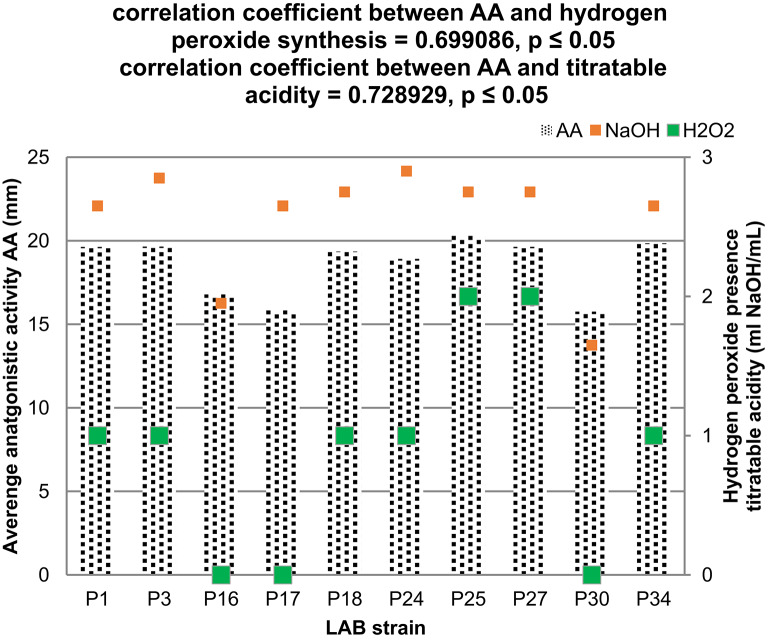
Correlation of the average antimicrobial activity (AA) of LAB strains with the ability to synthesize H₂O₂ and titratable acidity. Value 0 - no H_2_O_2_ production; value 1 - weak H_2_O_2_ production; value 2 - strong ability to synthesize H_2_O_2_. Pearson correlation analysis was performed using Statistica program, with a 95% confidence interval (*p* ≤ 0.05).

Figure 5 presents the ability of the tested strains to acidify the environment, expressed as titratable acidity. *Lb. plantarum* P1, P2, P24, P25, P27, P34, *Lb. brevis* P17, and *Lb. pentosus* P18 were the most effective in acidifying the environment, with titratable acidity values ranging from 2.65 to 2.90 mL NaOH/mL. In contrast, the remaining two strains classified as *Lb. brevis* (P16, P30) exhibited lower titratable acidity, with values ranging from 1.65 to 1.95 mL NaOH/mL.

In the Pearson analysis, a correlation between the average antimicrobial activity (AA) of the tested LAB strains and their capacity to synthesize H₂O₂ was demonstrated, with a correlation coefficient of 0.699086 at *p* ≤ 0.05 (Fig. 5). Similarly, a positive correlation was observed between AA and the ability of the tested strains to acidify the growth environment, with a correlation coefficient of 0.728929 at *p* ≤ 0.05. These findings indicate that both H₂O₂ synthesis and the production of acidic metabolites by the tested LAB strains play crucial roles in inhibiting the pathogenic bacteria used as test strains in this study.

## Discussion

Comparing the results of identification based on the 16S rRNA sequence and identification based on the fermentation profile, it can be concluded that bacteria belonging to the species *Lb. plantarum* provide a very good and consistent identification based on these methods. However, in the case of bacteria assigned to the species *Lb. brevis* based on the 16S rRNA sequence, identification based on the fermentation profile is divergent and often results in assignment to the species *Lb. pentosus* or, less frequently, to *Lb. plantarum*. Similar results of identification and convergence between methods were obtained by Zielińska et al.^29^. The conducted research confirms the necessity of 16S rRNA sequence analysis. All isolated LAB strains were catalase-negative, coagulase-negative, and classified as Gram-positive bacilli. They exhibited no hemolytic activity, indicating γ-hemolysis. It is important to note that both α-hemolysis and γ-hemolysis are non-pathogenic, while only β-hemolysis is linked to pathogenicity and may cause adverse effects in hosts, such as edema and anemia^18^.

Antibiotic resistance is an important feature of LAB. Zarzecka et al.^30^ indicate in their work that LAB may (but do not necessarily) act as a vector transmitting antibiotic resistance in the food environment. Research suggests that the acquisition of antibiotic resistance in LAB starter cultures is not a critical problem, but it raises concerns that cannot be ignored. The first step in in vitro research is to determine the phenotypic profile of antibiotic resistance of LAB strains. Of course, further research should include genotyping for antibiotic resistance due to the so-called silent genes, which are not expressed and do not manifest in the phenotype but can be transferred to other microorganisms^31^. In our research, in accordance with the EFSA guidelines^20^, we assessed the antibiotic resistance of the tested LAB strains towards nine antibiotics. These antibiotics belonged to two groups based on their mechanisms of action. The first group included antibiotics that limit the synthesis of the bacterial cell wall. In this group, we tested vancomycin, which belongs to glycopeptides, and ampicillin, which is a synthetic derivative of penicillin classified as a β-lactam antibiotic. At a concentration of 30 µg, all tested LAB strains were resistant to vancomycin. This fact is not surprising, as LAB, which until 2020 were classified under the genus *Lactobacillus* spp., are characterized by innate resistance, with resistance genes located on permanent elements of the genome and are not transferable^32^. However, when exposed to ampicillin at a concentration of 2 µg, 80% of the tested strains showed sensitivity to this antibiotic, while only 20% showed resistance. The obtained results are consistent with those of Coppola et al.^33^, who examined 63 strains of the *Lactobacillus rhamnosus* species isolated from food. They found that 100% of the strains were resistant to vancomycin, while none were resistant to ampicillin. The second group included antibiotics that inhibit the growth of susceptible microorganisms by blocking the synthesis of proteins, which is essential for cellular functions. This group encompassed tetracycline, streptomycin, kanamycin, gentamicin, chloramphenicol, erythromycin, and clindamycin. The tested LAB strains were 100% sensitive to tetracycline, chloramphenicol, and erythromycin, which is completely consistent with the results obtained by Coppola et al.^33^. For gentamicin, the same authors reported 98% resistant strains with a 2% proportion of sensitive strains. In our case, all strains (100%) were resistant to this antibiotic. Different results compared to the study by Coppola et al.^33^ were obtained for clindamycin. Specifically, 30% of the tested LAB strains were resistant to clindamycin, while 70% were assessed as moderately susceptible (MS). However, all tested *Lb. rhamnosus* strains described by Coppola et al.^33^ showed sensitivity to this antibiotic. These differences may result from the environment from which the strains were obtained and their taxonomic affiliation. In our research, the strains were obtained from the spontaneous fermentation of common onions, while the *Lb. rhamnosus* strains described by Coppola et al.^33^ were isolated from different samples of Parmigiano Reggiano cheese. *Lactobacillus* species are usually sensitive to chloramphenicol. In the case of the strains tested in this study, all strains belonging to the species: *Lb. brevis*,* Lb. plantarum*,* Lb. pentosus* were sensitive to this antibiotic. A high level of resistance to chloramphenicol was found in *Lb. delbrueckii* subsp. *bulgaricus* isolated from Chinese yogurt^31,34^. Many studies have demonstrated resistance of lactobacilli belonging to the *Lactobacillus* genus to aminoglycoside antibiotics such as kanamycin and streptomycin. Researchers explain this effect by the low permeability of external cellular structures^35–37^. Our studies also confirmed the resistance of the tested strains to kanamycin and streptomycin.

Mucin is the main component of the intestinal mucous layer. One of its roles is to protect the intestinal epithelium against undesirable, often pathogenic, microorganisms^38^. It is known that in germ-free animals (devoid of intestinal microbiota), large amounts of mucus accumulate in the intestinal lumen, which is not a desirable phenomenon^39^. One of the safety criteria for probiotic bacterial strains is their limited ability to hydrolyze mucin. In our research, we found that the isolated LAB strains were unable to grow when mucin was the sole carbon source in the culture medium. The OD_600_ value for such conditions ranged from 0.25 to 0.41, which is consistent with the research of Tarracchini et al.^22^. These researchers observed growth for *Lb. crispatus* strains in liquid MRS medium with mucin (without glucose), with OD_600_ values not exceeding 0.3.

The ability of probiotic bacteria to form biofilms is widely recognized as a beneficial trait. Biofilm formation is a fundamental, adaptable strategy that allows microorganisms to thrive in diverse environments^40^. Bacteria can develop biofilms on both biotic and abiotic surfaces in natural ecosystems as well as clinical settings^41^. Primarily, it facilitates colonization and prolonged persistence on intestinal mucosal surfaces. Additionally, biofilm formation acts as a protective barrier against pathogenic microorganisms through competitive interactions. However, it has been demonstrated that not all probiotic strains exhibit the ability to form biofilms on abiotic surfaces, such as glass or polystyrene^42^. According to FAO/WHO guidelines^17^, LAB strains intended for probiotic applications should exhibit high antimicrobial activity and a strong ability to form biofilms. Our study confirmed that all LAB isolates obtained from onions exhibited biofilm-forming capacity on both biotic and abiotic surfaces. Furthermore, they demonstrated high antimicrobial activity against foodborne contaminants. These findings align with the results of Aoudia et al.^43^, who reported the antimicrobial activity of *Lb. plantarum* WCFS1 a biofilm-forming strain, against *E. coli* O157:H7, *S.* Enteritidis, *S. aureus*, and *L. monocytogenes*. According to Bujňáková and Kmeť^44^, biofilm formation by LAB, expressed as absorbance measured for crystal violet extracted with alcohol, is considered low when absorbance values are below 0.1. In contrast, absorbance values between 0.2 and 0.3 indicate a strong biofilm-forming ability. Based on this scale, all strains isolated from onions exhibited a high capacity for biofilm formation on both abiotic and biotic surfaces. Our results (Table 4) confirm these findings. The BFC values of onion-derived LAB on abiotic surfaces (polystyrene plates) ranged from 1.37 to 5.47. The highest BFC value was observed in *Lb. plantarum* P24, which formed significantly stronger biofilms compared to other tested strains. In the remaining strains, BFC values did not exceed 3.5. On biotic surfaces (polystyrene plates coated with mucin), BFC values ranged from 0.65 to 1.54, with *Lb. plantarum* P1 exhibiting the highest BFC and *Lb. plantarum* P34 the lowest. Although biofilm formation on abiotic surfaces is not routinely tested in probiotic strains, strong adhesion ability is a crucial characteristic. Biofilm formation is a multi-stage process that enhances bacterial resistance to environmental stresses^45^. The hypothetical mechanism of probiotic action against pathogenic biofilms involves competitive interactions. Probiotic strains capable of biofilm formation compete with pathogenic microorganisms, potentially due to competition for nutrients and adhesion sites^46^.

The sensitivity of LAB to adverse environmental conditions varies and is often dependent on species and even the ecological niche from which they were isolated^47^. After ingestion, LAB strains encounter various stressors in the gastrointestinal tract, including unfavorable factors such as low pH and bile salts, as well as toxic metabolites of the intestinal microbiota. One such metabolite is phenol and its derivatives, primarily formed through the degradation of α-amino acids, tyrosine, and phenylalanine^47^. Phenol and its derivatives have a bacteriostatic effect; therefore, it is advisable to test the tolerance of potentially probiotic bacteria to this group of compounds. In our study, most LAB strains exhibited biomass production in the presence of phenol (0.3% *v/v*) at levels ranging from 25 to 50% of the maximum biomass observed in control samples. High tolerance to the presence of phenol in the growth environment of LAB was observed by Reuben et al.^27^. These researchers investigated the tolerance of LAB isolated from the gastrointestinal tract of healthy chickens to phenol. They found high tolerance of the tested bacteria to phenol at concentrations ranging from 0.1 to 0.3%, while phenol at a concentration of 0.4% was a factor that drastically reduced the number of LAB. A comparison of phenol tolerance between *Lb. plantarum* and *Lb. brevis* after 24 h revealed higher tolerance in *Lb. plantarum* strains. This observation aligns with the findings of Abouloifa et al.^48^, who studied the survival of *Lb. plantarum*, *Lb. brevis*, and *Lb. pentosus* isolated from fermented olives. Interestingly, for *Lb. brevis* strains P16, P17, and P30, we observed an increase in phenol tolerance after 72 h compared to 24 h. This suggests a potential adaptive response of these bacteria to chemical environmental stress. However, this effect was not observed in the other LAB species examined in this study. The obtained results confirmed the adverse effect of low pH levels (1.5–2.5) on the examined strains of LAB. Nevertheless, all tested LAB strains demonstrated high survivability at pH 3.0. From a technological point of view, LAB tolerance to high concentrations of sodium chloride is an important factor. Sodium chloride is commonly used in fermented foods to inhibit the growth of undesirable microorganisms, which are often introduced with raw materials. However, high salt concentrations negatively affect microbial survival by altering the osmotic pressure and decreasing water activity. In our study, LAB strains exposed to 10% (*w/v*) sodium chloride in MRS medium exhibited low tolerance to this condition. Similar results were reported by Reuben et al.^27^. The protection of bacteria against the effects of H₂O₂ is mainly achieved by catalase activity. Unfortunately, not all LAB are characterized by the presence of this enzyme. In the case of bacteria classified by 2020 as the genus *Lactobacillus*, this enzyme is not present^49^. H₂O₂ can exert dual effects in microbial environments: it can act as an inducer of reactive oxygen species (ROS) or as a constitutive, non-radical ROS. LAB strains can synthesize H₂O₂ as a non-radical ROS at approximately 350 ppm (0.035% w/v, 0.0149 M)^50^. In this process, H₂O₂ is synthesized by LAB through the catalytic activity of NADPH oxidase on the cell membrane and superoxide dismutase (SOD)^51–53^. In our study, LAB strains were exposed to an H₂O₂ concentration of 0.4% (*v/v*), equivalent to 0.1705 M, under aerobic conditions at an optimal growth temperature of 30 °C under aerobic conditions. The results revealed a significant reduction in biomass production, with growth levels dropping below 25% of the maximum absorbance observed in control conditions without H₂O₂. This decrease was statistically significant across all tested strains. However, analysis of Fig. 4 reveals that *Lb. brevis* strains (P16, P17, P30) exhibited markedly higher sensitivity to H₂O₂ exposure compared to *Lb. plantarum* and *Lb. pentosus* strains. Zotta et al.^54^ demonstrated that *Lb. plantarum* C17 tolerance to H₂O₂ depends on temperature and oxygen availability. Their findings indicated that at 35 °C under aerobic conditions, the strain tolerated H₂O₂ concentrations of up to 50 mmol/L, whereas under anaerobic conditions, tolerance decreased to 25 mmol/L. At 25 °C, *Lb. plantarum* C17 exhibited tolerance to 38 mmol/L H₂O₂ in aerobic conditions and 19 mmol/L under anaerobic conditions. These findings suggest that LAB tolerance to H₂O₂ depends not only on species but also on abiotic environmental factors.

Antagonistic activity is one of the most important probiotic features that should characterize strains claiming to be probiotics. One of the antimicrobial mechanism of LAB is the production of bacteriocins, which can inhibit biofilm synthesis and have potential as contraceptives^55^. An important feature of LAB in the context of antimicrobial activity is the ability to synthesize H₂O₂. H₂O₂ is known to be produced efficiently by LAB in anaerobic environments. Its antagonistic activity is directed against microorganisms that cannot decompose (they have a peroxidase deficiency). The main mechanisms of action of H₂O₂ against microorganisms are disruption of the proper metabolism of proteins, DNA and RNA, leading to blocking the processes of transcription and translation and, consequently, causing damage at the DNA level leading to cell death^56^. The antagonistic mechanism of action of lactic and acetic acids on the cells of pathogenic microorganisms is well known. It involves the diffusion of hydrogen ions H^+^ into the cell, which disturbs the cell’s metabolic processes, disturbs the permeability of cell membranes, and consequently leads to cell death^57^. Correlations between the average antagonistic activity and the synthesized acidic metabolites of LAB and the synthesis of H₂O₂ in bacteria belonging to the *Lb. acidophilus* species were demonstrated in the study of Klewicka and Libudzisz^58^. The presented studies also confirmed positive correlations of average antimicrobial activity with the synthesis of acidic metabolites and the synthesis of H₂O₂. Currently available statistical tools allow us to demonstrate that these are positive correlations. It can be concluded that the strains with high antimicrobial potential in the conducted study belong to the species *Lb. plantarum* (P1, P3, P24, P25, P27, P34 and *Lb. pentosus* (P18). Strains P16, P17, P30 belonging to the species *Lb. brevis* are characterized by lower antimicrobial potential towards pathogenic bacteria. This is related to lower acidification and no H₂O₂ synthesis.

## Conclusions

This study demonstrates that spontaneously fermented *Allium cepa* L. serves as a natural reservoir of diverse LAB, including *Lb. plantarum*, *Lb. pentosus*, and *Lb. brevis*, with promising probiotic potential. The isolated strains exhibited key probiotic characteristics, such as acid and bile tolerance, metabolic versatility, and antimicrobial activity against foodborne pathogens, which was primarily attributed to organic acid production, as indicated by titratable acidity, with H₂O₂ synthesis providing additional support. Their strong antimicrobial properties suggest potential applications in food preservation. Importantly, none of the tested strains demonstrated mucin hydrolysis, confirming their safety for probiotic use. These findings highlight fermented onion as a valuable source of LAB strains with beneficial properties, contributing to the development of functional foods. The isolation and characterization of novel LAB strains in this study mark a significant step in exploring unique microbial resources for food innovation. The selected strains exhibit promising traits, making them suitable candidates for further applications in the food industry.

Future research will focus on utilizing these strains to develop innovative preparations of osmotically dehydrated, fermented onion. This approach not only advances food processing technology but also creates new market opportunities, supporting the development of high-quality, probiotic-rich products. Overall, this work underscores the role of LAB in sustainable food production, aligning with consumer demand for functional and appealing foods.

## Electronic supplementary material

Below is the link to the electronic supplementary material.

Supplementary Material 1

## Data Availability

The research data supporting this study have been deposited in the Open Research Data Repository of Lodz University of Technology and are accessible at: https://doi.org/10.34658/RDB.INFQ5X.The nucleotide sequences of the isolated LAB strains *Lactiplantibacillus plantarum* P1, P3, P24, P25, P27, P34, *Levilactobacillus brevis* P16, P17, P30, and *Lactiplantibacillus pentosus* P18 have been deposited in the NCBI GenBank repository under the accession numbers PP733398, PP733399, PP733406, PP733407, PP733408, PP733410, PP733402, PP733403, PP733409, and PP733404, respectively. These datasets are publicly available at https://www.ncbi.nlm.nih.gov/genbank.

## References

[1] Elattar, M. M., Darwish, R. S., Hammoda, H. M. & Dawood, H. M. An ethnopharmacological, phytochemical, and pharmacological overview of onion (*Allium cepa* L.). *J. Ethnopharmacol. Eurostat.* 117779. https://ec.europa.eu/eurostat/statistics-explained/images/f/ff/Production_of_selected_vegetables_%28million_tonnes%2C_2022%29_30-10-2023.png (2024). 10.1016/j.jep.2024.117779 38262524

[2] Stoica, F., Rau, R. N., Velecu, I. D., Stănciuc, N. & Râpeanu, G. A comprehensive review on bioactive compounds, health benefits, and potential food applications of onion (*Allium Cepa* L.) skin waste. *Trends Food Sci. Technol.* **141**, 104173 (2023).

[3] Durmaz, L., Kiziltas, H., Karagecili, H., Alwasel, S. & Gulcin, İ. Potential antioxidant, anticholinergic, antidiabetic and antiglaucoma activities and molecular Docking of spiraeoside as a secondary metabolite of onion (*Allium cepa*). *Saudi Pharm. J.* **31**, 101760 (2023). 37693735 10.1016/j.jsps.2023.101760 PMC10485163

[4] Teshika, J. D. et al. Traditional and modern uses of onion bulb (*Allium Cepa* L.): a systematic review. *Crit. Rev. Food Sci.* **59**, 39–70 (2019). 10.1080/10408398.2018.1499074 30040448

[5] Perez-Gregorio, R. M., Garcıa-Falcon, M. S., Simal-Gandara, J., Rodrigues, A. S. & Almeida, D. P. Identification and quantificationof flavonoids in traditional cultivars of red and white onions at harvest. *J. Food Compos. Anal.* **23**, 592–598 (2010).

[6] Prakash, D., Singh, B. N. & Upadhyay, G. Antioxidant and free radical scavenging activities of phenols from onion (*Allium cepa*). *Food Chem.* **102**, 1389–1393 (2007).

[7] Vazquez-Armenta, F., Ayala-Zavala, J., Olivas, G., Molina-Corral, F. & Silva-Espinoza, B. Antibrowning and antimicrobial effects of onion essential oil to preserve the quality of cut potatoes. *Acta Aliment.* **43**, 640–649 (2014).

[8] Sagar, N. A., Pareek, S., Benkeblia, N. & Xiao, J. Onion (*Allium Cepa* L.) bioactives: chemistry, pharmacotherapeutic functions, and industrial applications. *Food Front.* **3**, 380–412 (2022).

[9] Saviano, G. et al. Metabolite variation in three edible Italian Allium cepa L. by NMR-based metabolomics: A comparative study in fresh and stored bulbs. *Metabolomics* **15**, 1–13 (2019). 10.1007/s11306-019-1566-6 31325058

[10] Shiomi, N., Benkeblia, N. & Onodera, S. The metabolism of the fructooligosaccharides in onion bulbs: A comprehensive review. *J. Appl. Glycosci*. **52**, 121–127 (2005).

[11] Ritsema, T., Smeekens, S. & Fructans Beneficial for plant and humans. *Curr. Opin. Plant. Biol.* **6**, 223–230 (2003). 12753971 10.1016/s1369-5266(03)00034-7

[12] Ferreira, V. C., Barroso, T. L. C. T., Castro, L. E. N. & Rosa, D. De Siqueira oliveira, L. An overview of prebiotics and their applications in the food industry. *Eur. Food Res. Technol.* **249**, 2957–2976 (2023).

[13] Grzelak-Błaszczyk, K., Czarnecki, A., Klewicki, R., Grzegorzewska, M. & Klewicka, E. Lactic acid fermentation of osmo-dehydrated onion. *Food Chem.* **399**, 133954 (2023). 36007442 10.1016/j.foodchem.2022.133954

[14] Thierry, A. et al. Microbial communities of a variety of 75 homemade fermented vegetables. *Front. Microbiol.* **14** (2023). 10.3389/fmicb.2023.1323424 PMC10757351 38163080

[15] Chen, X., Krug, L., Yang, M., Berg, G. & Cernava, T. The Himalayan onion (*Allium wallichii* Kunth) harbors unique spatially organized bacterial communities. *Microb. Ecol.* **82**, 909–918 (2021). 33723621 10.1007/s00248-021-01728-5 PMC8551121

[16] Bernal-Castro, C., Espinosa-Poveda, E., Gutiérrez-Cortés, C. & Díaz-Moreno, C. Vegetable substrates as an alternative for the inclusion of lactic acid bacteria with probiotic potential in food matrices. *J. Food Sci. Techno*. **61**, 833–846 (2024). 10.1007/s13197-023-05779-z PMC10933215 38487286

[17] Araya, M. et al. Ben embarek, P. Guidelines for the evaluation of probiotics in food. In *Joint FAO/WHO Working Group Report on Drafting Guidelines for the Evaluation of Probiotics in Food* 1–11 (Cordoba, 2002).

[18] Xu, Y. et al. Novel lactic acid bacteria with anti-hyperglycaemic properties: in vitro screening and probiotic assessment. *Food Biosci.* 105696 (2025).

[19] Song, Y. L. et al. Identification of and hydrogen peroxide production by fecal and vaginal lactobacilli isolated from Japanese women and newborn infants. *J. Clin. Microbiol.* **37** (9), 3062–3064 (1999). 10449509 10.1128/jcm.37.9.3062-3064.1999 PMC85459

[20] Rychen, G. et al. Guidance on the characterisation of microorganisms used as feed additives or as production organisms. *EFSA J.* **16**, 5206 (2018). 10.2903/j.efsa.2018.5206 PMC7009341 32625840

[21] Charteris, W. P., Kelly, P. M., Morelli, L. & Collins, J. K. Antibiotic susceptibility of potentially probiotic *Lactobacillus* species. *J. Food Prot.* **61**, 1636–1643 (1998). 9874341 10.4315/0362-028x-61.12.1636

[22] Tarracchini, C. et al. The core genome evolution of Lactobacillus crispatus as a driving force for niche competition in the human vaginal tract. *Microb. Biotechnol.* **16**, 1774–1789 (2023). 37491806 10.1111/1751-7915.14305 PMC10443340

[23] Naicker, P. R., Karayem, K., Hoek, K. G., Harvey, J. & Wasserman, E. Biofilm formation in invasive *Staphylococcus aureus* isolates is associated with the clonal lineage. *Microb. Pathog*. **90**, 41–49 (2016). 26546719 10.1016/j.micpath.2015.10.023

[24] Brink, M., Todorov, S. D., Martin, J. H., Senekal, M. & Dicks, L. M. T. The effect of prebiotics on production of antimicrobial compounds, resistance to growth at low pH and in the presence of bile, and adhesion of probiotic cells to intestinal mucus. *J. Appl. Microbiol.* **100**, 813–820 (2006). 16553737 10.1111/j.1365-2672.2006.02859.x

[25] Ilango, S., Pandey, R. & Antony, U. Functional characterization and microencapsulation of probiotic bacteria from *Koozh*. *J. Food Sci. Technol.* **53**, 977–989 (2016). 27162377 10.1007/s13197-015-2169-5 PMC4837729

[26] Jacobsen, C. N. et al. Screening of probiotic activities of forty-seven strains of *Lactobacillus* spp. By in vitro techniques and evaluation of the colonization ability of five selected strains in humans. *Appl. Environ. Microbiol.* **65**, 4949–4956 (1999). 10543808 10.1128/aem.65.11.4949-4956.1999 PMC91666

[27] Reuben, R. C., Roy, P. C., Sarkar, S. L., Alam, R. U. & Jahid, I. K. Isolation, characterization, and assessment of lactic acid bacteria toward their selection as poultry probiotics. *BMC Microbiol.* **19**, 1–20 (2019). 31718570 10.1186/s12866-019-1626-0 PMC6852909

[28] Tomusiak-Plebanek, A., Mruk, M., Rząca, S., Strus, M. & Arent, Z. Vitro assessment of anti-*Campylobacter* activity of *lactobacillus* strains isolated from canine rectal swabs. *BMC Vet. Res.* **18**, 112 (2022). 35317800 10.1186/s12917-022-03204-9 PMC8939066

[29] Zielińska, D., Rzepkowska, A., Radawska, A. & Zieliński, K. In vitro screening of selected probiotic properties of *Lactobacillus* strains isolated from traditional fermented cabbage and cucumber. *Curr. Microbiol.* **70**, 183–194 (2015). 25270682 10.1007/s00284-014-0699-0

[30] Zarzecka, U. & Zadernowska, A. Chajęcka-Wierzchowska, W. Starter cultures as a reservoir of antibiotic resistant microorganisms. *LWT-Food Sci. Technol.* **127**, 109424 (2020).

[31] Nunziata, L., Brasca, M., Morandi, S. & Silvetti, T. Antibiotic resistance in wild and commercial non-enterococcal lactic acid Bacteria and bifidobacteria strains of dairy origin: an update. *Food Microbiol.* **104**, 103999 (2022). 35287818 10.1016/j.fm.2022.103999

[32] Abriouel, H. et al. New insights in antibiotic resistance of *Lactobacillus* species from fermented foods. *Food Res. Int.* **78**, 465–481 (2015). 28433315 10.1016/j.foodres.2015.09.016

[33] Coppola, R. et al. Antibiotic susceptibility of *Lactobacillus rhamnosus* strains isolated from parmigiano Reggiano cheese. *Le Lait*. **85**, 193–204 (2005). 10.1016/j.femsle.2005.01.037 15727832

[34] Zhou, N. et al. Antibiotic resistance of lactic acid bacteria isolated from Chinese yogurts. *J. Dairy. Sci.* **95**, 4775–4783 (2012). 22916881 10.3168/jds.2011-5271

[35] Danielsen, M. & Wind, A. Susceptibility of *Lactobacillus* ssp. To antimicrobial agents. *Int. J. Food Microbiol.* **82**, 1–11 (2003). 12505455 10.1016/s0168-1605(02)00254-4

[36] Flórez, A. B. et al. Susceptibility of *Lactobacillus plantarum* strains to six antibiotics and definition of new susceptibility–resistance cutoff values. *Microb. Drug Resist.* **12**, 252–256 (2006). 17227210 10.1089/mdr.2006.12.252

[37] Muhammad, I. et al. Antibiotic resistance of probiotics isolated from Chinese corn Stover silage. *J. Appl. Anim. Res.* **51**, 102–114 (2023).

[38] Megur, A. et al. In vitro screening and characterization of lactic acid bacteria from Lithuanian fermented food with potential probiotic properties. *Front. Microbiol.* **14** (2023). 10.3389/fmicb.2023.1213370 PMC10516296 37744916

[39] Falk, P. G., Hooper, L. V., Midtvedt, T. & Gordon, J. I. Creating and maintaining the Gastrointestinal ecosystem: what we know and need to know from gnotobiology. *Microbiol. Mol. Biol. Rev.* **62**, 1157–1170 (1998). 9841668 10.1128/mmbr.62.4.1157-1170.1998 PMC98942

[40] Li, Y. et al. Biofilms formation in plant growth-promoting bacteria for alleviating agro-environmental stress. *Sci. Total Environ.* **907**, 167774 (2024). 37848152 10.1016/j.scitotenv.2023.167774

[41] Peng, Q., Tang, X., Dong, W., Sun, N. & Yuan, W. A review of biofilm formation of *Staphylococcus aureus* and its regulation mechanism. *Antibiot* **12**, 12 (2022). 10.3390/antibiotics12010012 PMC9854888 36671212

[42] Salas-Jara, M. J., Ilabaca, A., Vega, M. & García, A. Biofilm forming *Lactobacillus*: new challenges for the development of probiotics. *Microorganisms* **4**, 35 (2016). 27681929 10.3390/microorganisms4030035 PMC5039595

[43] Aoudia, N. et al. Biofilms of *Lactobacillus plantarum* and *Lactobacillus fermentum*: effect on stress responses, antagonistic effects on pathogen growth and Immunomodulatory properties. *Food Microbiol.* **53**, 51–59 (2016). 26611169 10.1016/j.fm.2015.04.009

[44] Bujňáková, D. & Kmeť, V. Functional properties of *Lactobacillus* strains isolated from dairy products. *Folia Microbiol.* **57**, 263–267 (2012). 22488103 10.1007/s12223-012-0121-x

[45] Probert, H. M. & Gibson, G. R. Bacterial biofilms in the human Gastrointestinal tract. *Curr. Issues Intest Microbiol.* **3**, 23–27 (2002). 12400635

[46] Tomé, A. R. et al. Use of probiotics to control biofilm formation in food industries. *Antibiot* **12**, 754 (2023). 10.3390/antibiotics12040754 PMC10135146 37107116

[47] Ghosh, S. & Pramanik, S. Structural diversity, functional aspects and future therapeutic applications of human gut Microbiome. *Archives Microbiol.* **203**, 5281–5308 (2021). 10.1007/s00203-021-02516-y PMC8370661 34405262

[48] Abouloifa, H. et al. Characterization of probiotic properties of antifungal *Lactobacillus* strains isolated from traditional fermenting green olives. *Probiot Antimicrob. Proteins*. **12**, 683–696 (2020). 10.1007/s12602-019-09543-8 30929140

[49] Derunets, A. S., Selimzyanova, A. I., Rykov, S. V., Kuznetsov, A. E. & Berezina, O. V. Strategies to enhance stress tolerance in lactic acid bacteria across diverse stress conditions. *World J. Microbiol. Biotechnol.* **40**, 126 (2024). 38446232 10.1007/s11274-024-03905-3

[50] Ito, A. et al. The screening of hydrogen peroxide-producing lactic acid bacteria and their application to inactivating psychrotrophic food-borne pathogens. *Curr. Microbiol.* **47**, 0231–0236 (2003). 10.1007/s00284-002-3993-1 14570275

[51] Bakavayev, S. et al. Cu/Zn-superoxide dismutase and wild-type like fALS SOD1 mutants produce cytotoxic quantities of H2O2 via cysteine-dependent redox short-circuit. *Sci. Rep.* **9**, 10826 (2019). 31346243 10.1038/s41598-019-47326-x PMC6658568

[52] Chen, L. et al. Metabolism of hydrogen peroxide by *Lactobacillus plantarum* NJAU-01: A proteomics study. *Food Microbiol,* **112** (2023). 10.1016/j.fm.2023.104246 36906310

[53] Sirokmány, G. & Geiszt, M. The relationship of NADPH oxidases and Heme peroxidases: fallin’ in and out. *Front. Immunol.* **10**, 394 (2019). 30891045 10.3389/fimmu.2019.00394 PMC6411640

[54] Zotta, T., Guidone, A., Ianniello, R. G., Parente, E. & Ricciardi, A. Temperature and respiration affect the growth and stress resistance of *Lactobacillus plantarum* C17. *J. Appl. Microbiol.* **115**, 848–858 (2013). 23782242 10.1111/jam.12285

[55] Fernandes, A. & Jobby, R. Bacteriocins from lactic acid bacteria and their potential clinical applications. *Appl. Biochem. Biotechnol.* **194**, 4377–4399 (2022). 35290605 10.1007/s12010-022-03870-3

[56] Mgomi, F. C., Yang, Y., Cheng, G. & Yang, Z. Lactic acid bacteria biofilms and their antimicrobial potential against pathogenic microorganisms. *Biofilm* **5** (2023). 10.1016/j.bioflm.2023.100118 PMC10139968 37125395

[57] Mussi, J. M. S. et al. M. Antagonistic activity of lactic acid bacteria isolated from Minas artisanal cheeses against *Brucella abortus*. *Braz J. Vet. Res. Anim. Sci.* **60** (2023).

[58] Klewicka, E. & Libudzisz, Z. Antagonistic activity of *Lactobacillus acidophilus* bacteria towards selected food-contaminating bacteria. *Pol. J. Food Nutr. Sci.* **13**, 169–174 (2004).

