# Supplementary material for: Spontaneously fermented Allium cepa L. as a source of lactic acid bacteria with probiotic potential
Source: Sci Rep. 2025 Jul 17;15:25970. doi: 10.1038/s41598-025-10037-7 (PMC12271377; doi:10.1038/s41598-025-10037-7)
Supplement: Supplementary file 1 — Supplementary Material 1 [file 41598_2025_10037_MOESM1_ESM.docx]

Table S1. Interpretation of antibiotic resistance results of tested bacteria based on growth inhibition zones in the disc test (Charteris et.al. 1998).

| **Antimicrobial agent** | | **Range of inhibition zone**  **[mm]** | | |
| --- | --- | --- | --- | --- |
|  |  | **R** | **MS** | **S** |
| **Group** | **Name (Abbreviation)**  **Concentration (µg)** |  |  |  |
| Group – 1: inhibitors of cells wall synthesis  (Glycopeptides)  (β-lactams, penicillins) | Vancomycin (VA) 30  Ampicilin (AMP) 2 | ≤14  ≤12 | 15-16  13-15 | ≥17  ≥16 |
| Group – 2: inhibitors of protein synthesis  (Tetracyclines)  (Aminoglycosides)      Single antibiotics  Macrolides  Lincosamide | Tetracycline (TE) 30  Streptomycin (S) 25  Kanamycin (K) 30  Gentamicin (CN) 10  Chloramphenicol (C) 30  Erythromycin (E) 5  Clindamycin (DA) 2 | ≤14  ≤11  ≤13  ≤12  ≤13  ≤13  ≤8 | 15-18  12-14  14-17  -  14-17  14-17  9-11 | ≥19  ≥15  ≥18  ≥13  ≥18  ≥18  ≥12 |

R- resistant, MS - moderately susceptible, S – sensitive

Table S2. Characteristics of lactic acid bacteria isolated from onions.

| **LAB strain** | **Type of Onion** | **Source** (Company/place of purchase) | **Gram straining** | **Catalase** | **Coagulase** | **Hemolytic activity** | **H_2_O_2_ production** |
| --- | --- | --- | --- | --- | --- | --- | --- |
| P1 | Shallot | Stokrotka (Maxima, Lithuania), Łódź, Poland | Purple, rod-shaped | - | - | - | 1 |
| P3 | Yellow | ALDI (ALDI Nord Group, Germany) Aleksandrów Łódzki, Poland | Purple, rod-shaped | - | - | - | 1 |
| P16 | Yellow | local vegetable market, Jastrzębia Góra, Poland | Purple, long rod-shaped | - | - | - | 0 |
| P17 | Yellow | POLOmarekt, Jastrzębia Góra, Poland | Purple,short rod-shaped | - | - | - | 0 |
| P18 | Red | POLOmarket Jastrzębia Góra, Poland | Purple, short rod-shaped | - | - | - | 1 |
| P24 | Yellow | local vegetable market, Poddębice, Poland | Purple, short rods, chains of 2–4 cells | - | - | - | 1 |
| P25 | Yellow | local vegetable market, Poddębice, Poland | Purple, rods in chains of 2 cells | - | - | - | 2 |
| P27 | Yellow | local vegetable market, Pabianice, Poland | Purple, rods in chains of 2 or 4 cells | - | - | - | 2 |
| P30 | Yellow | local vegetable market, Pabianice, Poland | Purple, rod-shaped | - | - | - | 0 |
| P34 | Red | local vegetable market, Pabianice, Poland | Purple, rod-shaped | - | - | - | 1 |

Value 0 - no H_2_O_2_ production; value 1 - weak H_2_O_2_ production; value 2 - strong ability to synthesize H_2_O_2_

Table S3. Phenotypic antibiotic sensitivity profile of tested lactic acid bacteria.

| **Antibiotic**  **[µg]** | **The average growth inhibition zone of** **LAB ± SD [mm]** | | | | | | | | | |
| --- | --- | --- | --- | --- | --- | --- | --- | --- | --- | --- |
|  | **P1** | **P3** | **P16** | **P17** | **P18** | **P24** | **P25** | **P27** | **P30** | **P34** |
| **VA 30** | 0.00±0.00 | 0.00±0.00 | 0.00±0.00 | 0.00±0.00 | 0.00±0.00 | 0.00±0.00 | 0.00±0.00 | 0.00±0.00 | 0.00±0.00 | 0.00±0.00 |
| **AMP 2** | 25.25±0.96 | 25.50±0.58 | 16.75±0.96 | 12.75±0.50 | 23.50±1.29 | 26.25±0.50 | 25.75±0.50 | 27.75±0.50 | 13.75±0.50 | 26.75±0.96 |
| **TE 30** | 25.00±0.82 | 23.00±0.82 | 22.75±0.50 | 22.75±0.50 | 22.75±0.50 | 21.50±0.58 | 21.75±0.50 | 22.50±0.58 | 22.00±0.82 | 22.00±0.82 |
| **S 25** | 0.00±0.00 | 0.00±0.00 | 0.00±0.00 | 0.00±0.00 | 0.00±0.00 | 0.00±0.00 | 0.00±0.00 | 0.00±0.00 | 0.00±0.00 | 0.00±0.00 |
| **K 30** | 0.00±0.00 | 0.00±0.00 | 0.00±0.00 | 0.00±0.00 | 0.00±0.00 | 0.00±0.00 | 0.00±0.00 | 0.00±0.00 | 0.00±0.00 | 0.00±0.00 |
| **CN 10** | 0.00±0.00 | 0.00±0.00 | 0.00±0.00 | 9.25±0.50 | 0.00±0.00 | 0.00±0.00 | 0.00±0.00 | 0.00±0.00 | 8.75±0.50 | 0.00±0.00 |
| **C 30** | 29.75±0.50 | 28.50±0.58 | 29.75±0.50 | 31.75±0.50 | 28.50±0.58 | 25.75±0.50 | 27.00±0.82 | 27.50±0.58 | 29.00±0.82 | 28.00±0.82 |
| **E 5** | 21.00±0.82 | 21.00±0.82 | 20.75±0.50 | 22.00±0.82 | 22.50±0.58 | 20.75±0.50 | 20.25±0.50 | 21.00±0.82 | 22.75±0.50 | 20.50±0.58 |
| **DA 2** | 9.50±0.58 | 10.75±0.50 | 8.00±0.00 | 8.00±0.00 | 10.50±0.58 | 8.50±0.58 | 9.25±0.50 | 8.75±0.50 | 7.75±0.50 | 8.75±0.50 |

VA – vancomycin; AMP – ampicillin; TE – tetracycline; S – streptomycin; K – kanamycin; CN – gentamicin; C – chloramphenicol; E – erythromycin; DA – clindamycin

| 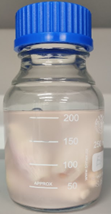 | 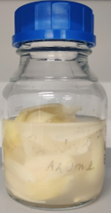 | 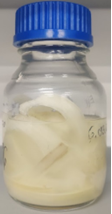 | 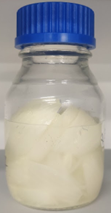 | 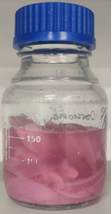 |
| --- | --- | --- | --- | --- |
| P1 | P3 | P16 | P17 | P18 |
| 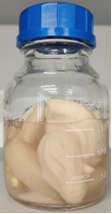 | 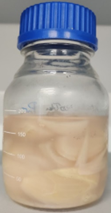 | 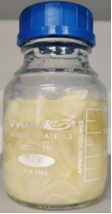 | 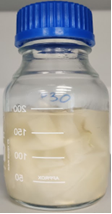 | 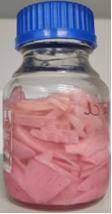 |
| P24 | P25 | P27 | P30 | P34 |

Figure S1. Fermented onion. The onion fermentation time: 9 days for *Lactiplantibacillus plantarum* P3, 11 days for *Lactiplantibacillus plantarum* P1, *Levilactobacillus brevis* P16, and P17, *Lactiplantibacillus pentosus* P18, 15 days for *Lactiplantibacillus plantarum* P24, and P25, 16 days for *Lactiplantibacillus plantarum* P27, P34, and *Levilactobacillus brevis* P30.

Figure S2. Growth of a) *Lactiplantibacillus plantarum* P1 b) *Lactiplantibacillus plantarum* P3 c) *Levilactobacillus brevis* P16 d) *Levilactobacillus brevis* P17 e) *Lactiplantibacillus pentosus* P18 f) *Lactiplantibacillus plantarum* P24 g) *Lactiplantibacillus plantarum* P25 h) *Lactiplantibacillus plantarum* P27 i) *Levilactobacillus brevis* P30 j) *Lactiplantibacillus plantarum* P34 in the presence of adverse environmental factors.
